# Advances in Forming Processes of Carbon Fiber-Reinforced Thermoplastic Composites: From Material Challenges to Interface Engineering

**DOI:** 10.3390/ma19142988

**Published:** 2026-07-10

**Authors:** Liran Sun, Shuo Wu, Donglong Chu, Tianshu Wang, Wei Shen, Zongan Li, Yongkang Fu, Wenbo Li, Shilong Xing

**Affiliations:** 1 School of Navigation and Shipping, Shandong Jiaotong University, Weihai 264209, China; sunliran124566@163.com (L.S.); wushuo84@163.com (S.W.); wangts163@163.com (T.W.); shenweizhenbang@163.com (W.S.);; 2 Shandong Avic Glory Aviation Standard Parts Co., Ltd., Jinan 250102, China; 3 School of Materials Science and Engineering, Shanghai Jiao Tong University, 800 Dongchuan Road, Shanghai 200240, China; sjtu_lwb@sjtu.edu.cn; 4 School of Construction Machinery, Shandong Jiaotong University, 5001 Haitang Road, Jinan 250357, China

**Keywords:** carbon fiber-reinforced thermoplastic composites, forming processes, mechanical properties, interface engineering

## Abstract

**Highlights:**

- Comprehensively reviews six processing routes with emphasis on defect control and recent progress.
- Proposes a novel tripartite framework for interface engineering: wettability improvement, path shortening, and interlayer functional design.
- Traces the evolution from commodity plastics to high-performance PAEKs and low-melting derivatives.

**Abstract:**

Carbon fiber-reinforced thermoplastic composites (CFRTPs) have attracted increasing attention in aerospace, transportation, marine engineering, and other advanced manufacturing fields owing to their high specific mechanical properties, impact resistance, weldability, reprocessibility, and potential recyclability. However, the high melt viscosity of thermoplastic matrices, the permeability limitations associated with different reinforcement architectures, and the chemical inertness of carbon fiber surfaces continue to restrict resin impregnation, interfacial bonding, defect control, and forming stability. This review systematically summarizes recent advances in CFRTP manufacturing from the perspective of material-derived processing challenges and interface engineering. First, representative thermoplastic matrix systems and reinforcement architectures are discussed, with emphasis on their effects on processability, crystallization behavior, resin flow, and load transfer. Subsequently, six major forming processes, including hot stamping, injection molding, pultrusion, filament winding, automated fiber placement, and additive manufacturing, are critically compared in terms of processing principles, typical defects, technical limitations, and application boundaries. Particular attention is given to process-induced quality issues such as voids, wrinkling, springback, fiber breakage, warpage, insufficient consolidation, and weak interlayer bonding. Finally, interface engineering strategies, including chemical surface modification, interfacial structural design, and functional interlayer design, are reviewed as practical routes to improve wetting, shorten impregnation pathways, and enhance fiber–matrix load transfer in high-viscosity thermoplastic systems. This review highlights that CFRTP manufacturing should be understood as a coupled materials–processing–interface problem rather than a single forming operation. Future development is discussed with emphasis on reproducible manufacturing, processability-oriented materials, scalable interface engineering, predictive modeling, and standardized structural validation.

## 1. Introduction

With the growing demand for lightweight, high-performance, and sustainable solutions in advanced manufacturing sectors such as aerospace, transportation, and marine engineering, material technology innovation has become a key driver of manufacturing upgrading. Traditional metallic materials like steel and aluminum alloys, while still dominant in structural strength and processability, are increasingly revealing intractable limitations in high-end manufacturing due to their high density, poor corrosion resistance, and complex forming and welding processes [1]. Against this backdrop, fiber-reinforced composites have been progressively introduced into high-end manufacturing. Their lightweight and high-strength properties [2,3,4,5], corrosion resistance [6,7], and excellent fatigue performance [8,9] have enabled the partial replacement of metals in structures such as aircraft skins and naval vessel masts, significantly reducing weight and extending service life [10]. Initially, fiber-reinforced composites were predominantly based on thermoset resin systems and were widely adopted in engineering applications. However, the irreversible curing of thermoset resins leads to long production cycles and difficulties in repair and recycling [11], making it challenging to meet the demands of large-scale and green manufacturing. Carbon fiber-reinforced thermoplastic composites, with their reprocessibility-enabled sustainability and potential for efficient industrial production, are emerging as important candidate materials for high-performance structural components [12,13,14].

**Figure 1:**
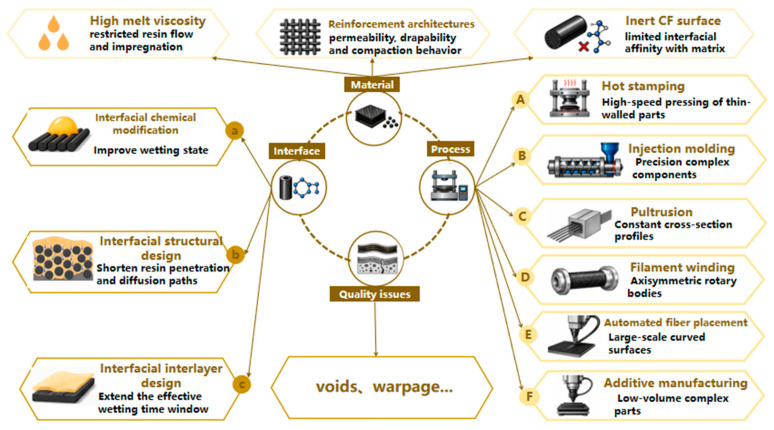
Organizational framework linking material-derived processing challenges, representative forming processes, interface engineering strategies, and forming quality issues in CFRTP manufacturing.

Recent studies have shown that forming processes [15,16,17] and interface engineering [18,19] have significant effects on the forming quality of CFRTP. As summarized in Table 1, previous reviews have provided valuable insights into specific forming routes and interfacial modification of thermoplastic composites, but these topics are usually discussed from relatively separate perspectives. There remains a need for further elucidation regarding how processing difficulties originating from material sources manifest across different forming processes, and how interfacial engineering can mitigate the consequent limitations in wetting and interfacial bonding. To address this issue, this paper first summarizes the thermoplastic matrix systems and reinforcement architectures, with emphasis on the processing difficulties caused by high melt viscosity, reinforcement configuration, and carbon fiber surface inertness. It then compares six representative forming processes, including hot stamping, injection molding, pultrusion, filament winding, automated fiber placement, and additive manufacturing, focusing on their typical quality issues, technical limitations, and application boundaries. Finally, interface engineering is discussed as a practical strategy to improve wetting, fiber impregnation, and interfacial bonding in high-viscosity thermoplastic systems. Through the organizational framework as illustrated in Figure 1, it is hoped that systematic theoretical foundations and technical references can be provided for the construction of an efficient, controllable, and sustainable composite forming system.

## 2. Overview of CFRTP

Composite materials are categorized based on their matrix and reinforcement. In CFRTP, the matrix serves as the core component that ensures structural integrity and functional integration. It governs load transfer and stress distribution through physical encapsulation of the reinforcement, while also determining the thermal resistance, interfacial bond strength, impact toughness, and processability of the composite. Additionally, the matrix provides environmental protection and enables functional customization. The reinforcement acts as the mechanical backbone of CFRTP. In particular, carbon fibers typically bears more than 90% of the applied load, thereby defining the stiffness and strength of the composite. The interface between the fiber and matrix functions as a bridge that controls load transfer efficiency and influences failure mechanisms through chemical bonding, mechanical interlocking, and molecular entanglement [25,26,27,28,29,30,31].

### 2.1. Development of Matrix Systems

Matrix selection has consistently been a pivotal factor influencing the performance and applicability of CFRTP [24]. Early systems predominantly utilized commodity thermoplastics such as polypropylene (PP) and polyamide (PA), which, due to their low cost and favorable processability, facilitated initial industrial adoption [32,33]. However, the inherent limitations of these materials including restricted thermal stability, high moisture absorption, and moderate mechanical properties have restricted their applications primarily to non-structural or semi-structural components, such as automotive interiors and consumer housings [34].

The increasing demands within the aerospace and advanced transportation sectors necessitate materials that not only possess high specific strength but also exhibit thermal resistance and long-term service stability. This shift in focus has led to significant advancements in amorphous high-performance thermoplastics, predominantly polyetherimide (PEI) and polyethersulfone (PES) [35,36]. PEI and PES are characterized by their excellent interfacial wettability, which facilitates their integration into advanced processing technologies such as automated fiber placement and additive manufacturing. The ability to be processed using such innovative techniques further enhances their appeal in industries where precision and performance are paramount [37,38]. Further developments have centered on semi-crystalline high-performance thermoplastics, particularly PPS, PEEK, and polyetherketoneketone (PEKK) [39,40]. Although PPS exhibits relatively lower toughness, its outstanding corrosion resistance, low moisture uptake, and ease of processing have led to its widespread use in automotive components, electronic packaging, and secondary aerospace structures. In contrast, PEEK and PEKK combine high glass transition temperatures with superior chemical resistance and mechanical toughness, enabling long-term service under extreme conditions encountered in aerospace and marine engineering applications [41,42,43].

**Figure 2:**
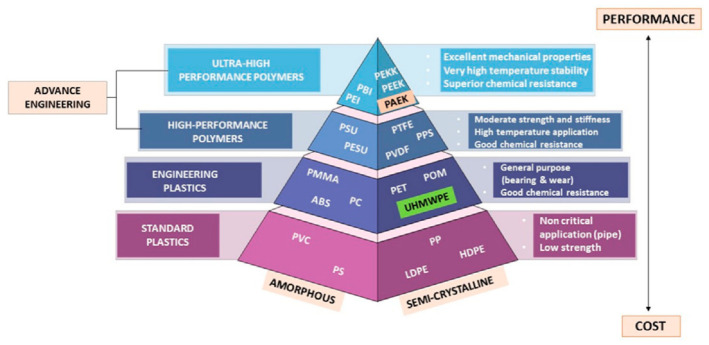
The hierarchy of amorphous and semi-crystalline thermoplastics [44].

The evolution of matrix systems in CFRTP generally progresses from low-cost commodity thermoplastics to high-performance amorphous polymers, and subsequently to semi-crystalline specialty engineering thermoplastics, as illustrated in Figure 2. This transition reflects the continuous escalation of performance requirements and is tightly interlinked with advances in processing technologies. Low-viscosity matrices such as PA and PP are well-suited for injection molding applications. Meanwhile, amorphous resins, including PEI and PES, exhibit enhanced compatibility with automated tape placement and high-temperature injection molding, attributed to their stable melt behavior and lack of crystallization tendencies. In contrast, high-melting-point, highly crystalline polymers such as PEEK and PEKK have emerged as the primary matrices for thermo-compression molding and automated fiber placement techniques. Table 2 summarizes representative CF/thermoplastic systems used in CFRTP prepregs or semi-finished products.

**Figure 3:**
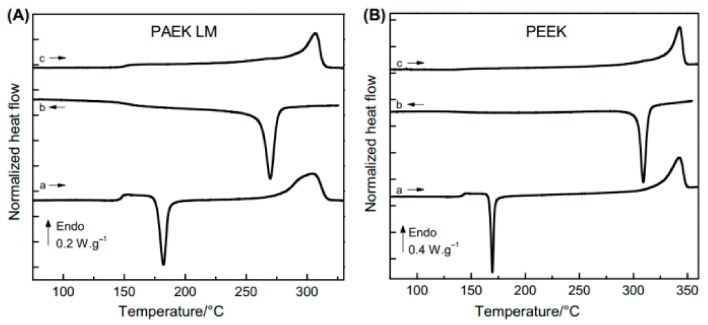
DSC thermograms of (**A**) LMPAEK and (**B**) PEEK: (a) first heating scan, (b) cooling scan, and (c) second heating scan [53].

Although the aforementioned thermoplastic matrices have been widely implemented in aerospace, automotive, and marine structures, their intrinsically high melting temperatures and melt viscosities continue to impose substantial processing constraints, particularly in the fabrication of large-scale or geometrically complex components [50,51,52]. To mitigate the high melt viscosity of PEEK, which restricts deposition rate and narrows the processing window, Victrex developed a low-melting polyaryletherketone (LMPAEK), subsequently commercialized by Toray Advanced Composites in the form of TC1225 prepreg. Jérémie Audoit et al. [53] comparatively investigated the thermal behavior of LMPAEK and conventional PEEK. Differential scanning calorimetry (DSC) analysis shown in Figure 3 indicated that LMPAEK exhibits a significantly lower melting temperature than PEEK, corresponding to a reduction of approximately 37 °C, while maintaining a comparable glass transition temperature about 150 °C. For automated fiber placement processes, the reduction in melting temperature directly lowers the required processing temperature, thereby decreasing the heating power demand of the consolidation source. Alternatively, under identical heating conditions, faster melting can be achieved, enabling higher layup speeds and improved manufacturing efficiency. In this context, the reduction in the effective processing window afforded by LMPAEK plays a pivotal role, as it enhances the applicability of advanced composites in demanding engineering applications. This advancement in material design is crucial not only for optimizing production rates but also for minimizing energy consumption during manufacturing processes, thus aligning with sustainability goals in the composites industry.

Following the marked improvement in processability, increasing attention has been directed toward the service performance of low-melting PAEK systems and their comparison with conventional PAEK matrices. Moraes et al. [40] evaluated the influence of LMPAEK and PEEK matrices on the static mechanical properties of carbon fiber-reinforced composites. The results indicated comparable strength and stiffness for both systems. For T300 carbon fiber-reinforced laminates, the tensile strengths of CF/PEEK and CF/LMPAEK were 567 MPa and 578 MPa, respectively, while the corresponding tensile moduli were 42.3 GPa and 40.4 GPa. Pereira et al. [54] further investigated the high-velocity impact behavior of thermoplastic composites based on APC AS4D/PEKK-FC and Toray TC1225 LMPAEK T700G using high-energy dynamic impact testing. The TC1225 LMPAEK T700G system exhibited a penetration threshold velocity approximately 50% higher and a penetration energy threshold more than 100% greater than those of the APC AS4D/PEKK-FC composite. Moreover, the onset velocity for detectable delamination in TC1225 LMPAEK T700G was approximately twice that of the PEKK-based laminate, indicating superior resistance to high-velocity impact damage for the LMPAEK-based composite system.

**Figure 4:**
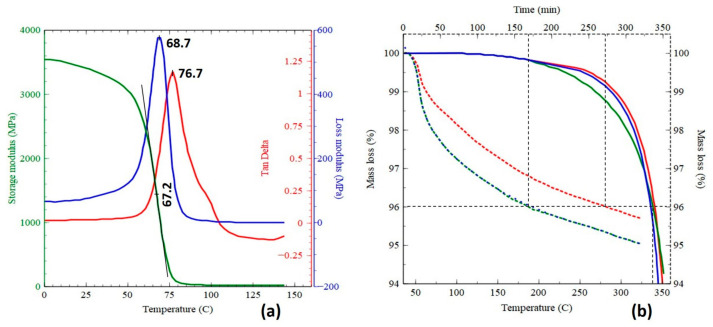
Thermoset (TS) matrix characteristics: (**a**) dynamic mechanical analysis (DMA) results; (**b**) thermal gravimetric analysis (TGA) results [55].

To reconcile processability for complex geometries with high mechanical performance, Adumitroaie et al. [55] proposed a hybrid strategy combining thermosetting and thermoplastic matrices. As shown in Figure 4a, a low-viscosity thermosetting epoxy resin was first used to achieve high-quality impregnation of continuous fibers, producing carbon fiber-reinforced thermoset (CFRTS) filaments. During the subsequent printing process, the pre-impregnated CFRTS filament was fed into the printer head and embedded into a simultaneously extruded molten thermoplastic matrix, resulting in a continuous fiber-reinforced thermoset–thermoplastic composite. The low viscosity of the thermoset ensured effective fiber wet-out, shown in Figure 4b. Meanwhile, thermogravimetric analysis indicated only ~4% mass loss at approximately 340 °C under short-term exposure, demonstrating adequate thermal stability for processing. The thermoplastic phase provided appropriate melt viscosity and processing window control during extrusion through the printing nozzle. Compared with single-matrix thermoset or thermoplastic systems, the continuous fiber-reinforced thermoset–thermoplastic additive manufacturing approach enabled improved impregnation quality and exhibited competitive tensile and compressive performance.

### 2.2. Reinforcement

Apart from the matrix, the reinforcement architecture is equally critical for CFRTP performance. The performance of CFRTP is strongly governed by the geometric configuration and spatial architecture of the reinforcement. The reinforcement not only defines the mechanical response of the composite, but also influences interlaminar stability and processing adaptability. Accordingly, maximizing the structural efficiency of the reinforcement has become a key focus in CFRTP research [56]. Carbon fibers are generally classified according to their continuity into continuous and discontinuous forms. Continuous fibers are typically employed in one-dimensional (1D), two-dimensional (2D), or three-dimensional (3D) architectures, whereas discontinuous reinforcements are commonly introduced as chopped fibers or nonwoven carbon fiber mats [57].

In 1D reinforcement architectures, all continuous fibers are aligned in a single direction, and no transverse reinforcing elements are present between fiber bundles or within the inter-bundle regions. As a result, the load-bearing capacity is primarily mobilized along the fiber axis [58]. Continuous unidirectional carbon fiber tapes are the representative form of 1D reinforcement and remain the most extensively utilized architecture in high-performance CFRTP structural components. The continuous alignment of fibers in a single direction enables maximum specific strength and specific modulus, meeting the demand for directional reinforcement along principal load paths in large-scale structures. Moreover, the regular architecture and uniform resin distribution of such tapes promote stable interfacial consolidation during melt processing, ensuring strong compatibility with continuous manufacturing techniques, including automated tape placement, pultrusion, and filament winding [59].

**Figure 5:**
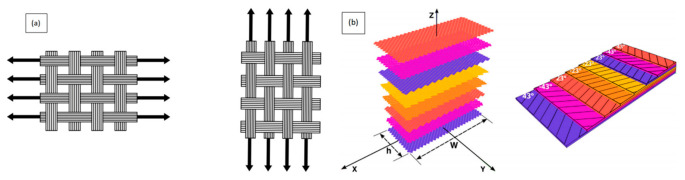
(**a**) 2D reinforcement [60], (**b**) laminated reinforcement [58].

In 2D reinforcement architectures, fibers are arranged along two or more in-plane directions, as illustrated in Figure 5a,b [58,60]. The most representative form is woven fabric, in which warp and weft yarns are interlaced within the plane. Such architectures provide improved in-plane quasi-isotropy and enhanced deformability, making them suitable for near-net-shape processes such as thermo-compression molding and thermoforming. The intrinsic inter-yarn porosity within woven fabrics serves as resin flow channels, facilitating uniform impregnation and reducing void content after consolidation. Compared with continuous unidirectional tapes, woven reinforcements exhibit lower sensitivity to layup orientation, consolidation path, and applied pressure during processing, resulting in a broader processing window. This makes them particularly advantageous for manufacturing complex curved shells and medium-scale structural components [61,62,63,64].

**Figure 6:**
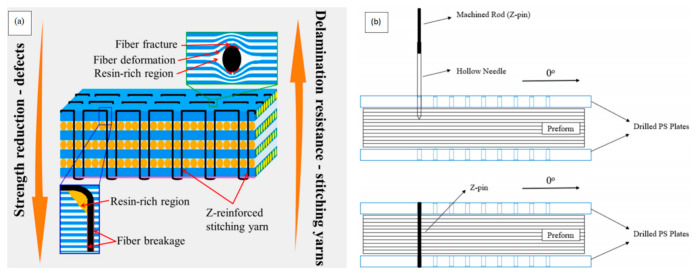
(**a**) Stitching reinforcement [65]. (**b**) Z-Pin reinforcement [66].

Because two-dimensional reinforcements lack through-thickness fiber constraint and load-bearing elements, their interlaminar interfaces are prone to delamination under complex loading or impact conditions. To enhance interlaminar mechanical performance, Z-pinning and stitching techniques have been introduced. By incorporating through-thickness reinforcing elements, these approaches significantly improve interlaminar shear strength and peel resistance, as illustrated in Figure 6a,b [65,66]. However, such methods inevitably disrupt in-plane fiber continuity and local architectural uniformity, thereby compromising in-plane load-bearing capacity. In addition, through-thickness reinforcements may induce thermal bridging effects, reducing thermal insulation performance and limiting their suitability for high-temperature structural applications [67,68]. Against this backdrop, integrally formed 3D reinforcement architectures have gained increasing attention. Compared with conventional 2D woven fabrics, 3D structures offer several advantages, including structural continuity, near-net-shape capability, high design flexibility, substantially improved interlaminar shear performance, and enhanced impact and fatigue resistance [69,70,71,72].

Chopped carbon fibers or hybrid reinforcements, which consist of a blend of long and short fibers and are characterized by their limited fiber length and random orientation, are highly suitable for blending with thermoplastic pellets for subsequent injection molding, extrusion, or compression molding processes. Their primary benefits include excellent flowability, short cycle times, and high mold adaptability, facilitating the efficient mass production of geometrically intricate components. Although their reinforcement efficiency is inferior to that of continuous fiber systems, optimizing the fiber length distribution, volume fraction, and orientation can achieve relatively high mechanical performance while preserving processing efficiency. Consequently, these systems find widespread application in automotive, electronics, and structural joining components [73,74].

Nonwoven carbon fiber mats are composed of randomly oriented fibers that are consolidated into a three-dimensional network through needling, hot pressing, or wet-lay processes. These structures exhibit high resin permeability and compressibility, making them suitable for thermo-compression molding, in situ consolidation, and continuous belt processing. They can serve as primary reinforcements or be co-consolidated with woven fabrics or tapes to enhance interlaminar toughness and impact resistance. With the advancement of recycled carbon fiber technologies, nonwoven mat reinforcements have emerged as a promising approach that combines mechanical performance with sustainability considerations [75,76].

### 2.3. Material-Derived Processing and Interfacial Challenges

The material advantages of CFRTP are often accompanied by corresponding processing challenges. Unlike thermoset composites, CFRTP require the thermoplastic matrix to be heated to a molten or highly viscous state during forming, so that it can impregnate fiber bundles and interlaminar regions within a limited processing time. This issue is particularly pronounced for high-performance thermoplastics such as PEEK, whose high melt viscosity restricts resin flow and readily leads to dry spots, voids, and local resin-rich or fiber-rich regions. Increasing temperature, pressure, or consolidation time can improve impregnation to some extent, but may also induce matrix thermal degradation, fiber damage, residual stress accumulation, and dimensional instability. For semi-crystalline thermoplastic matrices, cooling rate and thermal history further affect crystallinity and crystalline morphology, thereby causing fluctuations in interlaminar properties, toughness, and dimensional stability. Therefore, CFRTP forming requires a careful balance among forming quality and manufacturing efficiency [77,78,79].

Reinforcement architecture further increases the complexity of this balance. Continuous unidirectional tapes provide high load-bearing efficiency, but their tightly packed fiber bundles increase the resin impregnation distance and reduce local permeability. Woven fabrics, three-dimensional reinforcements, and chopped fibers can improve formability or provide different resin flow paths, while they may also introduce tow waviness, fiber misalignment, inter-bundle voids, and local thickness non-uniformity. Therefore, the challenge in CFRTP manufacturing is not simply to melt the matrix, but to achieve sufficient flow, impregnation, consolidation, and crystallization control within a limited and material-dependent processing window [80,81].

In addition to matrix viscosity and reinforcement architecture, the fiber–matrix interface represents a material-derived limitation that directly affects both processing quality and mechanical performance. Carbon fibers generally possess chemically stable graphitic surfaces and relatively low surface energy, whereas many high-performance thermoplastic matrices show limited wettability and molecular compatibility with untreated carbon fibers. This makes it difficult to establish sufficient interfacial bonding during short forming cycles. Weak interfacial bonding reduces load transfer efficiency and promotes fiber pull-out, interfacial debonding, and delamination under mechanical loading [23].

These material-derived challenges cannot be fully eliminated by process parameter adjustment alone. Higher temperature, higher pressure, or longer consolidation time may improve impregnation or interlayer bonding in specific cases, but they also increase the risk of thermal degradation, fiber disturbance, residual stress, longer cycle time, and higher manufacturing cost. Therefore, CFRTP forming should be understood as a coupled materials–process problem. The following section discusses how these intrinsic material challenges manifest differently in representative forming processes, while the subsequent interface engineering section considers practical strategies for improving wetting, shortening effective impregnation pathways, and enhancing fiber–matrix load transfer in high-viscosity thermoplastic systems.

## 3. Advances in Processing

The processing of CFRTP is crucial for structural applications, as it determines mechanical properties, dimensional accuracy, and interfacial bonding quality, which ultimately dictate service reliability [22,24]. As discussed above, the material characteristics of CFRTP give rise to various processing difficulties, which manifest in different forms during forming. Based on differences in processing form and load transfer mode, existing CFRTP forming methods can be broadly divided into two categories: re-forming of semi-finished products and direct melt forming. The former uses prepreg tapes, sheets, or fabrics as intermediate forms and achieves rapid shaping through processes such as stamping and automated fiber placement. The latter directly forms components from the molten state through injection molding, pultrusion, filament winding, and additive manufacturing, thereby enabling both structural complexity and manufacturing continuity [20,82,83,84]. This section reviews these six representative processes, focusing not only on their processing principles, but also on their technical limitations and application boundaries.

### 3.1. Stamping

Stamping is one of the most widely adopted and mature processing methods for thermoplastic composites. In this process, prepreg laminates or sheets are initially heated to the melting temperature of the thermoplastic matrix, followed by rapid transfer into the mold for compression forming and subsequent cooling. Due to the melt-processable nature of thermoplastic resins and their significantly reduced viscosity at elevated temperatures, the entire forming cycle can typically be completed within 10–90 s, substantially shorter than that of conventional thermoset composite processing. Benefiting from its short cycle time, high consolidation quality, and ability to achieve high fiber volume fractions, stamping is particularly well-suited for the mass production of medium-sized components with relatively simple geometries. In recent years, it has been widely applied in aerospace and automotive structures and is gradually expanding into lightweight ship structures [20]. However, during stamping, the material undergoes complex thermo-mechanical and rheological coupling under transient high-temperature and rapid cooling conditions, leading to defects such as fiber slippage, wrinkling, and dimensional deviations, which significantly compromise the quality of structural components.

**Figure 7:**
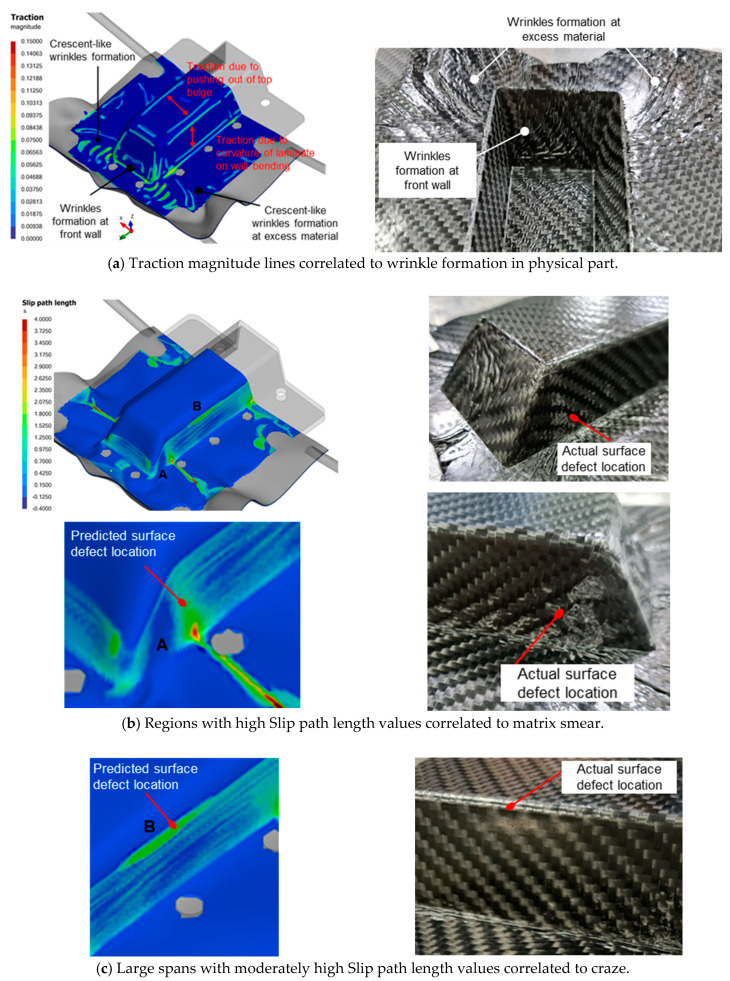
Correlation between simulated indicators (traction magnitude and slip path length) and observed defects in thermoformed parts [81].

Researchers commonly employ a combination of numerical simulation and experimental validation to investigate the control of defects such as fiber slippage and wrinkling. Behrens et al. [85] studied the influence of different layup orientations and clamping strategies on the forming quality of a scaled battery housing for a plug-in hybrid vehicle through combined experimental and numerical analyses. Their results showed that a 45/45° layup effectively mitigates wrinkling and fiber breakage caused by restricted shear deformation, exhibiting better geometric adaptability. In contrast, for the 0/90° layup, wrinkling occurred in the tunnel region due to limited shear deformation, resulting in a local thickness increase to 4.5 mm, nearly three times the original value, far exceeding the mold gap of 1.5 mm and ultimately leading to fiber fracture. However, the transferability of the research results to geometrically scaled-up or real production environments has not been validated. Tan et al. [81] further proposed a defect-prediction framework using slip path length and traction force as indicators. As shown in Figure 7, the simulated traction force and slip path length correlated well with observed surface defects. Their results indicate that the forming rate is the dominant factor affecting defect formation. When the forming speed increased from 25 mm/s to 80 mm/s, the areas with high slip path length and high traction force expanded by approximately 15% and 10%. The bending stiffness in the material model was not calibrated through independent experiments.

**Figure 8:**
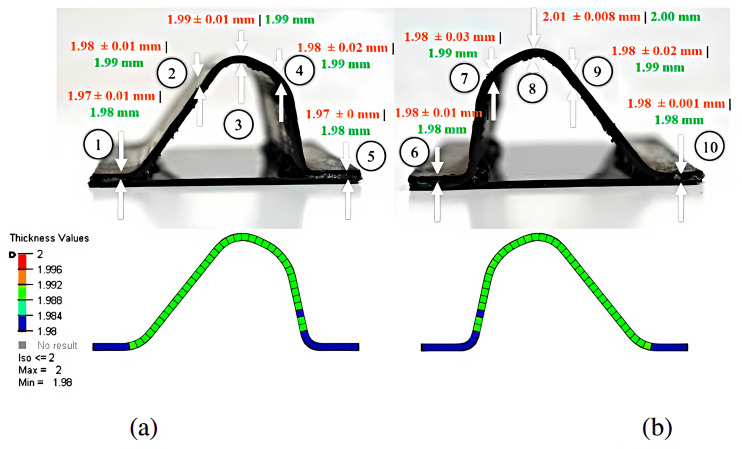
Thickness variation in the hat structure: (**a**) Side 1, (**b**) Side 2 represented as (upper) measured average thickness ± standard deviation and (lower) predicted thickness variation (uniform along z-direction) [87].

To address dimensional deviation, Hwang et al. [86] investigated the springback behavior of woven carbon fiber/polycarbonate composites after hot stamping of V-shaped structures and found that the springback angle depended strongly on both the layup and forming angle. For a [(±45)]_6_ layup, the springback angles of 60° and 90° V-shaped parts were approximately 2.60° and 1.72°, respectively, whereas for a [(0/90)]_6_ layup, the corresponding values were about 2.38° and 1.19°. Limaye et al. [87] established a manufacturing-response pathway for thermoformed hat-shaped structures and found that the formed parts exhibited a measurable thickness gradient, with the thickness ranging from 1.97 to 2.01 mm. As shown in Figure 8, the measured thickness distribution agreed well with the simulation results. More importantly, a 3% thickness variation increased the deformation under transverse compression, shear, and bending by 16.73%, 16.43%, and 16.80%, respectively, while reducing the peak impact load from 12 to 11.3 kN. Xu et al. [88] explored a hybrid metal–composite laminate strategy for CF/PA6-7003 aluminum structures and showed that an Al-CF-Al configuration reduced springback to 0.37 mm, which was much lower than that of pure metal (2.63 mm) and CF-Al-CF configurations (0.79 mm). Although this study provides directions for process optimization, the material model is oversimplified.

**Figure 9:**
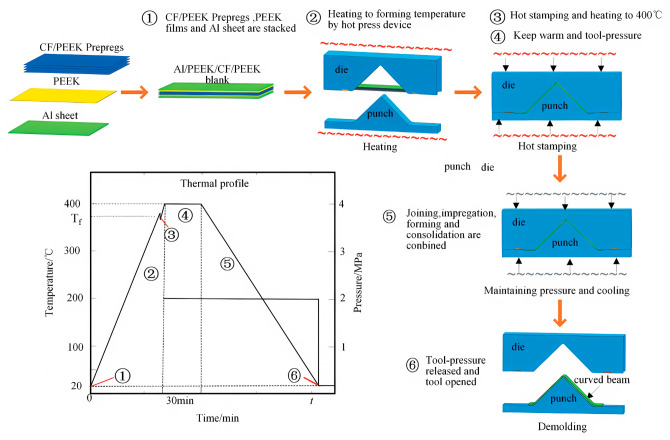
The one-step hot stamping forming process route [89].

**Figure 10:**
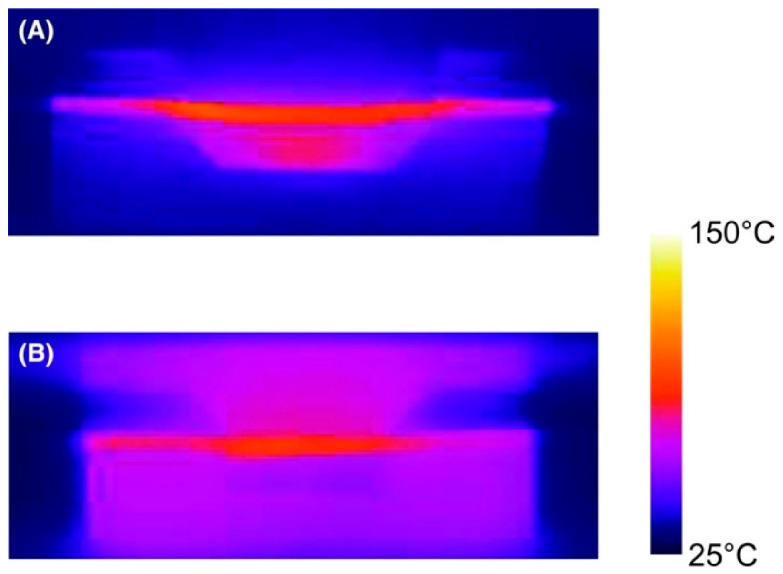
Thermal images of the carbon fiber-reinforced plastic (CFRP) composite parts and the zirconia molds. (**A**) K of the zirconia molds is ~3.3 W/mK. After 20 s microwave irradiation. (**B**) K of the zirconia molds is ~3.9 W/mK. After 260 s microwave irradiation [91].

In terms of process optimization, Lu et al. [89] proposed a one-step hot stamping process, as shown in Figure 9, in which heating, impregnation, shaping, and consolidation were integrated into a single stamping operation. By sequentially stacking aluminum sheets, PEEK films, and CF/PEEK prepregs and then conducting simultaneous heating and stamping in a heated mold, the process produced high-quality V-shaped beams with a springback angle below 0.2°, thickness reduction below 0.2 mm, curved beam strength of 195 N·mm/mm, and interlaminar strength of 16.5 MPa. This integrated route simplifies the manufacturing procedure and shows potential for automated production, although its applicability has only been demonstrated for relatively simple 90° V-shaped beams. Kang et al. [90] employed a mold integrated with an induction coil to generate heat directly within the laminate, enabling rapid heating at up to 40 °C/min and cooling at 20 °C/min. The fabricated CF/PPS composites exhibited a porosity below 1%, a tensile strength of 1912.57 ± 262.58 MPa in the 0° direction, and an interlaminar shear strength of 73.92 ± 0.41 MPa. This process shortens the molding cycle from hours to minutes, greatly improving production efficiency and energy use while maintaining good mechanical properties. But it lacks validation of temperature field uniformity. Shimamoto et al. [91] proposed microwave-assisted ceramic-mold stamping, in which carbon fibers acted as microwave-sensitive media and zirconia molds with low thermal conductivity reduced heat loss and microwave shielding. Experimental results showed that only 20 s of microwave irradiation was required to heat the CF/PP laminate to a formable temperature of 80 °C, and bending forming could be completed within 1 min. The lack of mechanical performance evaluation limits the assessment of its structural applicability. Figure 10 presents infrared thermography comparing microwave heating behavior using zirconia molds with different thermal conductivities.

The studies summarized above indicate that most stamping-induced defects originate from the mismatch between rapid macroscopic forming and insufficient local deformation accommodation. Fiber slippage occurs when local mold curvature, clamping constraints, or non-uniform pressure cause non-uniform movement of fiber tows or plies. Wrinkling forms when the required in-plane deformation cannot be adequately accommodated, and the excess material deformation is released as out-of-plane instability. After forming, non-uniform cooling, crystallization shrinkage, and other factors further induce springback or geometrical deviations. Although numerical models and defect indicators have improved the prediction of local deformation and thickness changes, their general applicability remains limited because most validations are still based on specific material systems and mold geometries. From an industrial perspective, thermo-stamping offers one of the most attractive routes for high-throughput CFRTP manufacturing, but its broader application to large or geometrically complex load-bearing structures requires more robust control of local temperature fields, deformation modes, and cooling histories.

### 3.2. Injection Molding

Injection molding involves melting and plasticizing the material within a barrel, followed by high-pressure injection into a closed mold and subsequent cooling to yield the final part. Due to its high efficiency, precise dimensional control, and high degree of automation, it stands as one of the most representative processes for short carbon fiber-reinforced thermoplastic composites. This process is characterized by short cycle times and is particularly well-suited for small-to-medium-sized components with intricate geometries and stringent dimensional tolerances. However, the mechanical performance of molded parts is significantly influenced by fiber length, orientation, and distribution, and is generally inferior to that of continuous fiber composites. At present, performance enhancement in injection-molded parts is primarily constrained by two issues: fiber breakage and orientation control during mold filling, as well as shrinkage and warpage during cooling and solidification.

**Figure 11:**
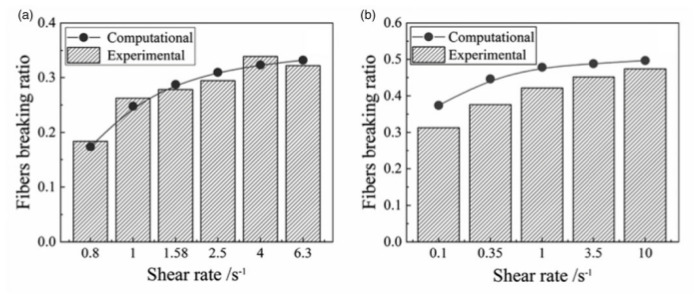
The breaking ratio of fiber with different initial lengths at specific shear rates was calculated and compared with the experimental results. (**a**) The initial length of fiber is 3 mm and (**b**) the initial length of fibers is 6 mm [92].

**Figure 12:**
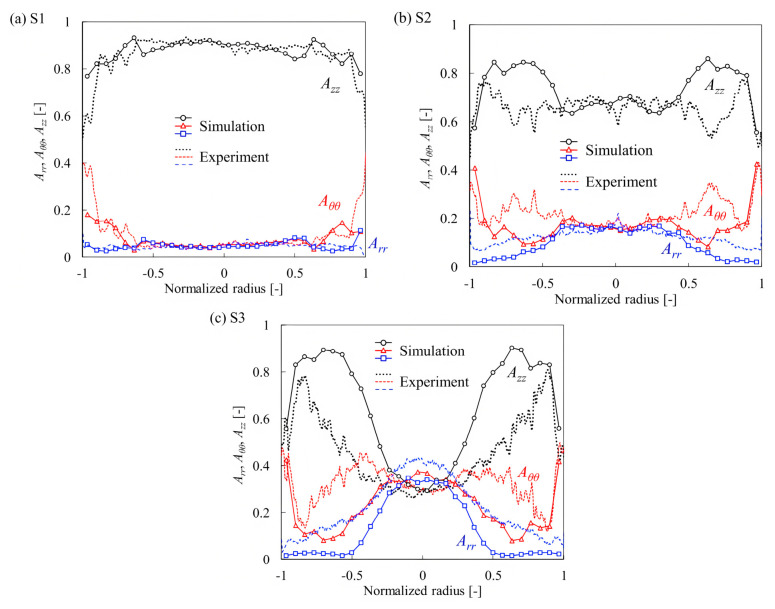
Profile of the orientation tensor components along the radius at (**a**) S1, (**b**) S2, and (**c**) S3 in the sprue obtained from the experiment and the simulation [93].

Fiber breakage and orientation evolution are central to the performance of injection-molded short-fiber composites. To clarify the fracture behavior of fibers in a shear flow field, Kang et al. [92] developed a shear-induced buckling model based on Oseen’s disturbance theory and the Euler buckling criterion. Their results showed that fiber breakage is governed by the combined effects of fiber aspect ratio and shear rate: longer fibers are more prone to buckling, while higher shear rates increase the axial compressive stress imposed by the melt flow. Experimental validation using GF/PP further confirmed that the fiber breaking ratio increased with both shear rate and initial fiber length. Figure 11 compares the predicted and measured fracture ratios for fibers with different initial lengths. Sasayama et al. [93] used particle-level simulation combined with μCT measurements to predict the orientation distribution of short fibers during injection molding. Their results showed that fiber orientation varies strongly with the local flow field: fibers aligned along the flow direction near the sprue inlet (S1) became more randomly oriented in the sprue bottom (S3) region (as shown in Figure 12) and then mainly reoriented in the circumferential direction after entering the disk. The study also indicated that fiber length affects orientation evolution, with shorter fibers showing a lower degree of flow direction alignment. Although both studies were validated using GF rather than CF systems, they provide useful mechanistic references for understanding shear-induced fiber breakage and flow-induced orientation evolution in injection-molded CFRTP.

**Figure 13:**
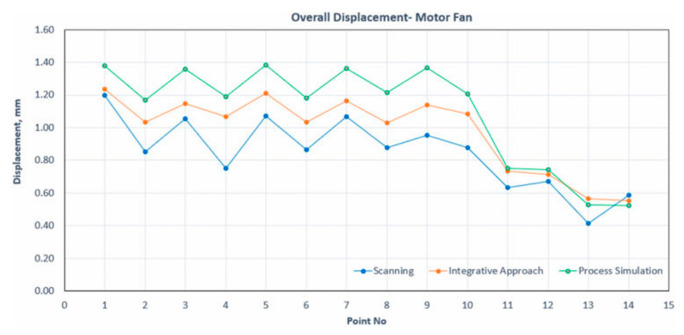
Warpage on reference points for scanning, process simulation, and integrative approach for motor fan [95].

Shrinkage and warpage during injection molding are key defects affecting dimensional accuracy and shape stability. To improve prediction accuracy, Li et al. [94] proposed a multi-layer model based on core-skin structure for crystalline parts in rapid thermal cycling molding, taking into account the differences in crystallinity and elastic modulus of each layer due to different thermal histories and shear histories. This model divides the injection-molded product into multiple independent layers with different crystal morphologies and mechanical properties along the thickness direction. The average error between the predicted and experimental values under this model can be reduced to 3.5–7.0%. Similarly, Divekar et al. [95] showed that neglecting material nonlinearity, viscoelasticity, and PVT behavior at high cooling rates is a major source of warpage prediction error. By introducing a temperature-dependent nonlinear thermo-mechanical model, their integrated simulation approach improved warpage prediction accuracy by 6–12%. Figure 13 compares the warpage displacement of a motor fan obtained by 3D scanning, integrated simulation, and conventional simulation.

**Figure 14:**
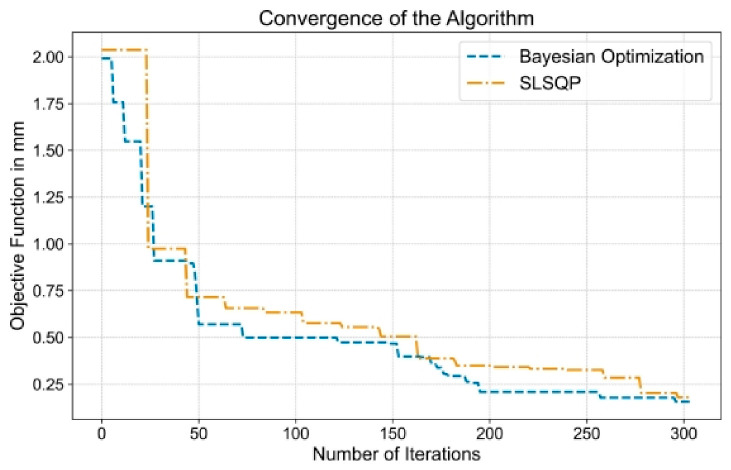
Convergence of the Bayesian optimization algorithm and the sequential least squares programming (SLSQP) algorithm. On the y-axis, the objective function value is shown, which measures the average distance of the deformed part to the desired part. On the x-axis, the number of iterations is shown [96].

Recent studies have further shifted from passive warpage prediction to active compensation and control. Tillmann et al. [96] proposed a cavity-shape compensation strategy based on Bayesian optimization and Gaussian process regression. In this approach, the cavity geometry was modified through free-form deformation, and the surrogate model was used to search for a compensated cavity that could offset the predicted post-cooling deformation. As shown in Figure 14, this strategy reduced the average deviation between the molded and target shapes from about 2.0 mm to below 0.5 mm, demonstrating the potential of automated cavity compensation. However, the optimization still requires a relatively large number of iterations, resulting in a comparatively high computational cost. Li et al. [97] combined a back-propagation neural network with a genetic algorithm to optimize warpage in fiber-reinforced composite injection molding. The neural network established the nonlinear relationship between fiber/process parameters and warpage, while the genetic algorithm searched for the parameter combination that minimized deformation. This approach reduced the maximum warpage from 0.1939 mm to 0.1020 mm, but the training of the neural network relies on a limited set of orthogonal experiment samples, resulting in limited generalization ability.

For injection-molded CFRTP, the key challenge is not simply whether the molten matrix can fill the mold cavity, but whether the fiber length, orientation, interfacial bonding, and crystallization behavior can be controlled during rapid plasticization, filling, packing, and cooling. The strong shear field that promotes mold filling may also shorten carbon fibers and generate a pronounced skin-core orientation gradient, leading to anisotropic mechanical properties. Meanwhile, non-uniform temperature and pressure histories can cause uneven crystallization, shrinkage gradients, residual stresses, and warpage. These issues are strongly coupled: fiber orientation affects local stiffness and shrinkage, while voids or weak interfacial bonding reduce the efficiency of load transfer. Therefore, the evaluation of injection-molded CFRTP should move beyond simple indicators such as fiber content or average strength, and should instead clarify how processing parameters are translated into fiber-scale evolution, defect formation, and part-level performance.

### 3.3. Pultrusion

Pultrusion represents a continuous and highly efficient manufacturing technique for producing thermoset or thermoplastic composites, particularly well-suited for fabricating long structural profiles with uniform cross-sections. In this process, continuous carbon fiber tows or fabrics are initially impregnated with a thermoplastic resin. The impregnated reinforcement is subsequently drawn through a sequence of dies under a constant tensile force. During this stage, the material undergoes heating and melting within a heating die, followed by compaction and shaping in a forming die, and is then cooled and solidified to achieve the desired profile. Finally, the consolidated composite profile is continuously extracted from the die system. Compared with batch forming processes, pultrusion offers high productivity, good dimensional repeatability and low material waste, making it particularly suitable for large-scale production of straight structural components such as rods, beams, and stiffeners [98,99].

Unlike thermosetting resins, which generally have low viscosities before curing, thermoplastic melts exhibit much higher viscosities during processing. This makes it difficult for the resin to rapidly penetrate the compact microstructure of fiber bundles within the limited residence time inside the die. As a result, dry spots, voids, and non-uniform resin distribution are often formed, especially at high fiber volume fractions or high pulling speeds. Once these defects are generated in the heated die, they are difficult to eliminate in the subsequent compaction stage and may extend continuously along the pultruded profile. Therefore, current research on thermoplastic pultrusion mainly focuses on improving impregnation through process parameter optimization, die design, and alternative low-viscosity processing routes [21,100].

**Figure 15:**
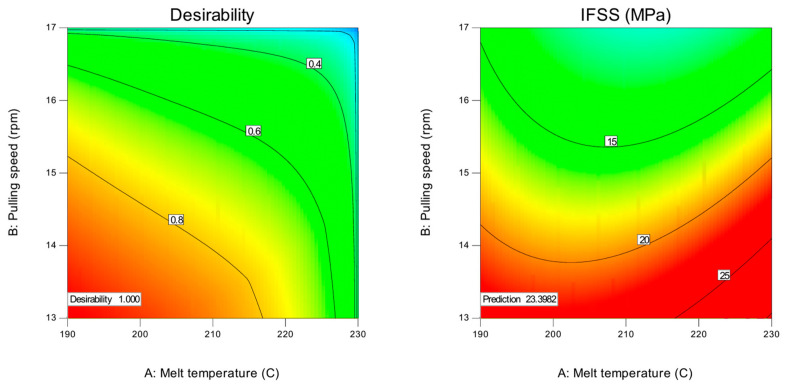
Optimization results of the extrusion–pultrusion process based on the Box–Behnken response surface methodology [3].

In terms of process parameter optimization, Tipboonsri et al. [101] investigated the influence of melting-zone temperature, thermal-reduction-zone temperature, and pulling speed on impregnation quality. Their results showed that increasing the die temperature and reducing the pulling speed were beneficial for resin penetration. The optimal processing conditions were a melting-zone temperature of 230 °C, thermal-reduction-zone temperatures of 160/150 °C, and a pulling speed of 10 cm/min, under which the unimpregnated ratio was reduced to 8.03%. Budiyantoro et al. [3] optimized four processing parameters in an extrusion–pultrusion process using the Box–Behnken response surface methodology, including melt temperature, pulling speed, the number of spreading pins, and fiber treatment. The results indicated that fiber treatment was the most critical factor affecting interfacial bonding. In particular, liquid nitrogen treatment maximized the surface roughness of carbon fibers, thereby significantly improving the degree of impregnation. As shown in Figure 15, the optimal parameter combination obtained from the Box–Behnken response surface analysis was a melt temperature of 190 °C, a pulling speed of 40 cm/min, three spreading pins, and liquid nitrogen treatment. Under these conditions, the interfacial shear strength reached 24.2 MPa, while both impregnation quality and forming stability were maintained. It should be noted that the pulling speeds used in these optimized processes are still far below the economically viable production speeds required for industrial thermoplastic pultrusion. This suggests that parameter optimization alone is insufficient to fully resolve the trade-off between impregnation quality and production efficiency.

**Figure 16:**
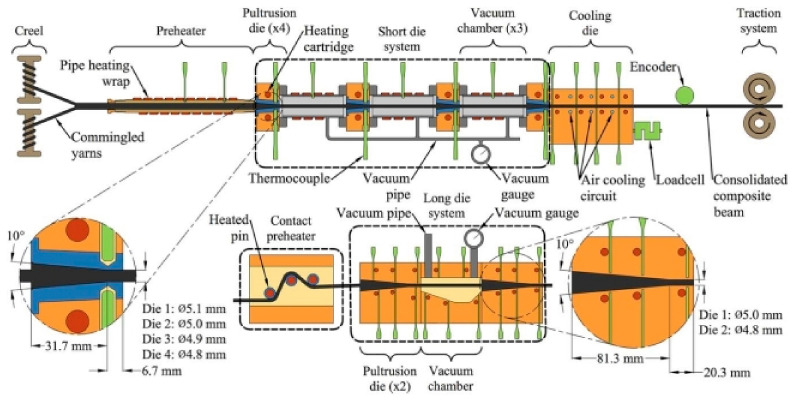
Schematic cross-section view of the lab scale pultrusion apparatus used during the experiments [102].

**Figure 17:**
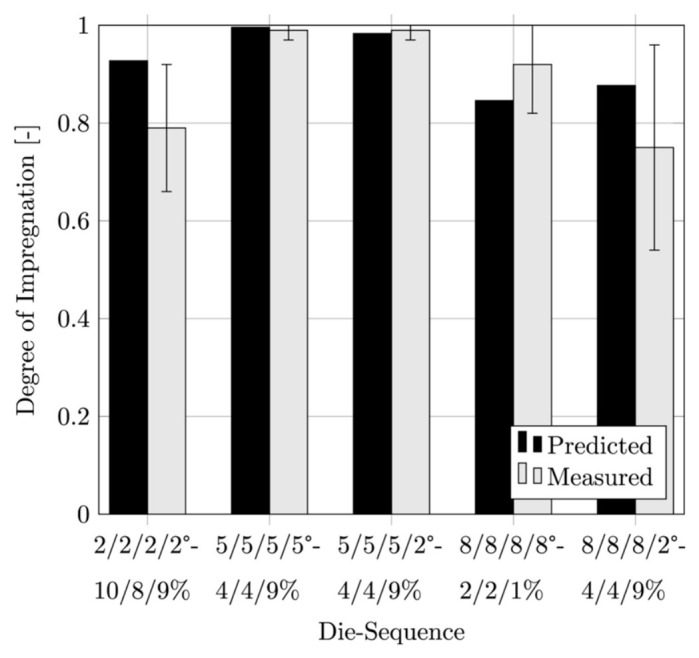
Degree of impregnation measured and predicted for the different die sequences tested. Error bars represent plus or minus one standard deviation [103].

In terms of die design, researchers have sought to improve impregnation by increasing the pressure difference between the fiber bundles and the molten resin. Lapointe et al. [102] designed a multi-die vacuum-assisted pultrusion system for carbon/PEEK commingled yarns, in which several short dies were arranged in series and separated by heated vacuum chambers, as shown in Figure 16. The multi-die configuration improved impregnation mainly by allowing the material to remain longer at the target temperature and under impregnation pressure. Compared with single-die pultrusion, it reduced the void content by more than 4 percentage points on average, increased the in-plane shear strength by approximately 29 MPa, and lowered the average pulling force by about 240 N. The best rods were obtained using four dies, vacuum assistance, a pulling speed of 50 mm/min, and a die temperature of 400 °C, with a void content of 1.3%. Lessard et al. [103] further shifted die design from empirical adjustment to model-assisted optimization by comparing multi-die sequences with different taper angles and outlet area reduction ratios. As shown in Figure 17, the 5° taper-angle sequences provided a better balance between impregnation quality and pulling force than the 2° and 8° configurations, and their interlaminar shear strength was approximately 10% higher than that of the other samples. These studies demonstrate the potential of multi-die systems to broaden the processing window of thermoplastic pultrusion, although their effectiveness remains dependent on precursor architecture and relatively low validated pulling speeds.

**Figure 18:**
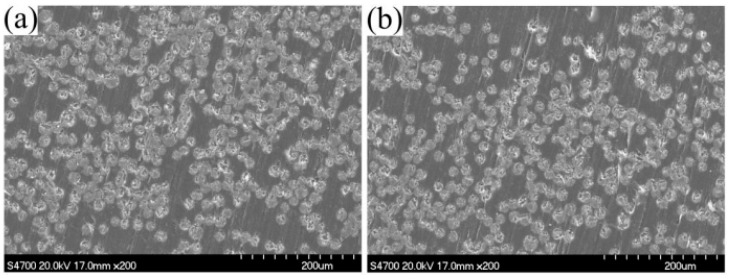
SEM micrographs of the polished composites with injection chamber at (**a**) 90 °C and (**b**)100 °C [104].

**Figure 19:**
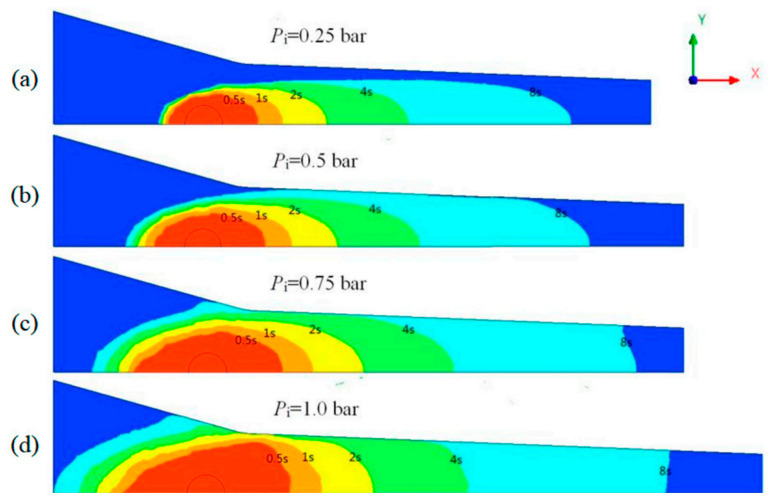
Flow front distribution of resin at different time with different injection pressures: (**a**) Pi = 0.25 bar; (**b**) Pi = 0.5 bar; (**c**) Pi = 0.75 bar; (**d**) Pi = 1.0 bar [105].

Beyond parameter optimization and die design, some studies address the impregnation limitation of high-viscosity thermoplastic melts by employing low-viscosity monomer systems in reactive pultrusion (RIM). Chen et al. [104] developed a thermoplastic reactive pultrusion line using ε-caprolactam (viscosity ≈ 5 mPa·s) as the monomer, which polymerizes in situ via anionic ring-opening polymerization inside the die to form a PA6 matrix. Their work mainly examined the effects of injection chamber temperature and die heating-zone temperature on impregnation and composite performance. The results showed that, when the injection chamber temperature was controlled at 90–100 °C, excellent impregnation was achieved and the composite cross-sections were nearly void-free, as illustrated by the SEM images in Figure 18. In addition, the highest interlaminar shear strength of 71.5 MPa was obtained at a die heating-zone temperature of 180 °C, representing a 13.7% increase compared with 170 °C. Ding et al. [105] simulated the impregnation of ε-caprolactam monomer (viscosity 50 mPa·s) in a conical impregnation chamber using the finite volume method, analyzing the effects of pulling speed and injection pressure on impregnation time and resin backflow distance. When the injection pressure was 0.5 bar, the complete impregnation time was 5.60 s. As the pulling speed increased from 0 to 20 cm/min, the impregnation time decreased slightly from 5.60 to 5.15 s. However, when the speed further increased to 80 cm/min, the impregnation time increased to 7.20 s due to higher radial flow resistance within the fiber bundles. Meanwhile, higher pulling speeds significantly suppressed resin backflow, reducing the backflow distance to 15.1 mm at 80 cm/min. Figure 19 illustrates the evolution of the resin flow front in the impregnation chamber under different injection pressures. While RIM bypasses the high-viscosity issue of thermoplastic melts, it demands more in terms of mold corrosion resistance, exotherm management, and polymerization kinetics.

Existing studies have shown that process parameter optimization, multi-die systems, and reactive low-viscosity monomer routes can alleviate the impregnation limitations of thermoplastic pultrusion to some extent. Nevertheless, these strategies must be evaluated in terms of the trade-off among impregnation quality, process complexity, and production efficiency. Process optimization often relies on relatively low pulling speeds, while multi-die and vacuum-assisted systems impose stricter requirements on thermal control and pulling force management. Reactive pultrusion can bypass the high viscosity of thermoplastic melts, but it shifts the processing challenge toward exotherm control and the chemical compatibility between the monomer/polymer system and the tooling. Therefore, future research should not only aim to further improve impregnation, but also to maintain stable consolidation under higher pulling speeds and more scalable processing conditions. Particular attention should be paid to simplified and optimized die systems, coupled thermal-flow-reaction control, and process windows that are transferable across different matrix systems, precursor architectures, fiber volume fractions, and profile geometries.

### 3.4. Filament Winding

**Figure 20:**
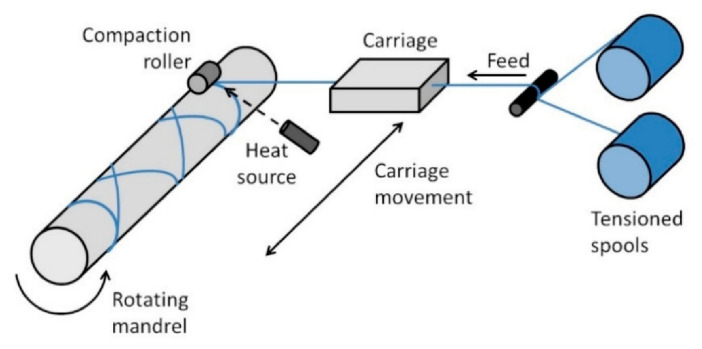
Filament winding with in situ consolidation [106].

Filament winding involves winding continuous fibers onto a rotating mandrel under controlled tension along predetermined angles and trajectories, creating a layered structure through either prepreg or in situ impregnation with thermoplastic resin. Figure 20 depicts the schematic of an in situ consolidation filament winding process. For thermoplastic composites, the tapes are typically heated in-line using laser, hot gas, or infrared sources to melt the resin and achieve interlayer bonding. After winding, the structure is cooled and removed from the mandrel to obtain dense and dimensionally stable hollow components. This process provides high forming precision, efficient fiber utilization, and flexible control over fiber placement angles. This process offers the advantages of high automation and relatively low cost, making it suitable for manufacturing high-performance rotational bodies, such as high-pressure gas cylinders, deep-sea pipelines and drive shafts.

**Figure 21:**
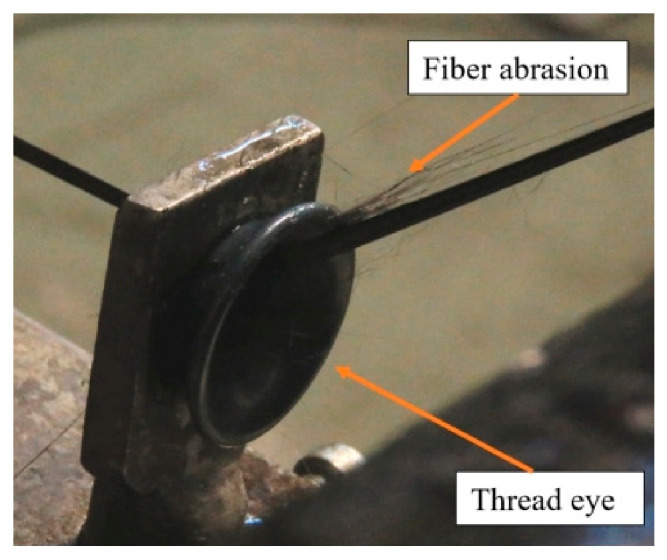
Fiber abrasion at the winding eye at 80 N [107].

A central issue in filament winding is the coupled control of fiber tension, winding speed, and consolidation quality. Fiber tension directly affects fiber alignment, compaction, fiber volume fraction, and residual stress, while improper tension may lead to fiber slippage, surface abrasion, or local geometric distortion. Błachut et al. [107] showed that increasing the winding tension from 3 N to 80 N raised the fiber volume fraction from 45% to 63%, increased the axial tensile strength from 2205 MPa to 3087 MPa, and improved the burst pressure of composite vessels from 170 bar to 205 bar. These results indicate that higher tension can enhance compaction and load-bearing capacity. Nevertheless, excessive tension also caused severe fiber abrasion at the guide eye, as shown in Figure 21, and increased the sensitivity of fiber placement on curved mandrel surfaces.

**Figure 22:**
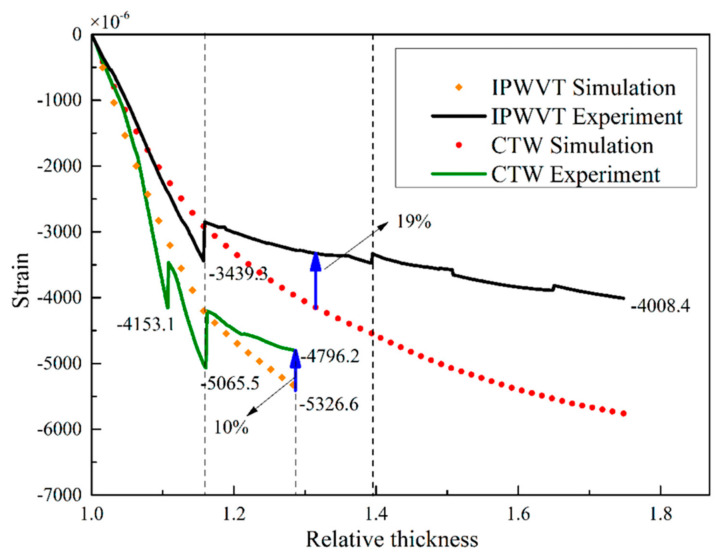
Comparison between online strain monitoring results and simulation results [108].

**Figure 23:**
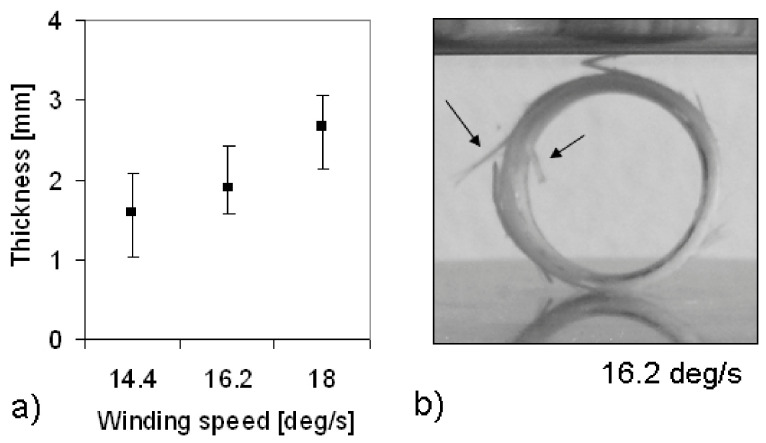
(**a**) Effect of the winding speed on the composite thickness and (**b**) appearance of a sample wound at 16.2 °/s [110].

To improve tension stability, recent studies have moved from constant-tension winding toward variable and feedback-based control strategies. Chen et al. [108] proposed an in-plane variable tension winding strategy for a composite hub, in which gradient tension was applied along different axial positions to reduce local overstress at the hub edge. Compared with constant-tension winding, this strategy increased the initial failure speed of the rotor by 160%, as shown in Figure 22. This result demonstrates the potential of spatially tailored tension control, although its long-term service reliability still requires further validation. In thermoplastic winding, tension regulation is also closely coupled with thermal history. Geng et al. [109] reported that winding speed was the most influential parameter affecting the tensile strength of laser-assisted CF/PPS composites. Excessively high speeds may cause insufficient heating and weak interlayer bonding, whereas excessively low speeds may overheat the matrix and promote excessive resin flow under compaction. Quadrini et al. [110] provided direct evidence that winding speed affects thickness and surface morphology in laser-assisted thermoplastic winding. At 14.4 °/s, the low viscosity of the polypropylene matrix led to interlayer interpenetration and fiber extrusion, while increasing the speed to 18 °/s maintained the matrix in a more suitable semi-solid state and produced a composite thickness close to the target value of approximately 2.6 mm, as shown in Figure 23. Xu et al. [111] further developed a three-stage tension control strategy based mainly on speed regulation, where the main drive roller speed was adjusted to stabilize winding tension. This provides an indirect route for tension control, although the strategy was developed under the assumption of symmetric filament winding and remains mainly validated at laboratory scale.

Real-time monitoring and adaptive feedback provide another route to stabilizing the winding process. Mindermann et al. [112] developed a robotic impregnation end-effector equipped with fiber tension sensors and established a closed-loop control system linking tow tension with the motion of the robotic end-effector. During high-risk operations such as hooking, the tool center point speed could be automatically reduced to approximately 75 mm/s, thereby suppressing sudden tension fluctuations and reducing the risk of uneven impregnation or fiber damage. This work highlights the value of process feedback in complex winding trajectories. Still, online tension does not directly represent the final prestress state in the consolidated component, which may be affected by resin flow, cooling shrinkage, stress relaxation, and mandrel removal. Consequently, tension monitoring needs to be combined with structural sensing or post-process validation when prestress or residual stress is a critical design parameter.

**Figure 24:**
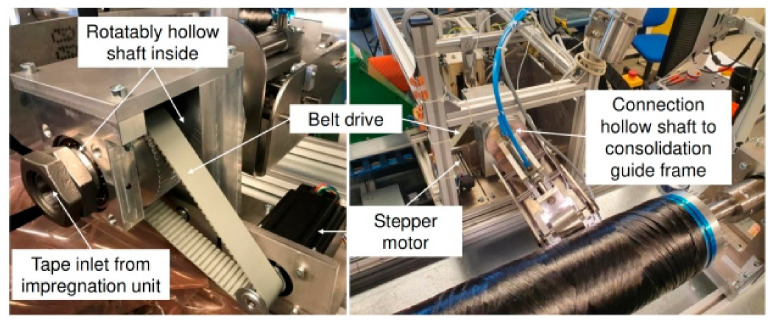
Winding angle realization based on the construction of a rotation axis of the consolidation unit by means of a stepper motor with holding torque, belt drive with torque transmission via rotatably mounted hollow shaft and mechanical connection via consolidation guide frame [113].

**Figure 25:**
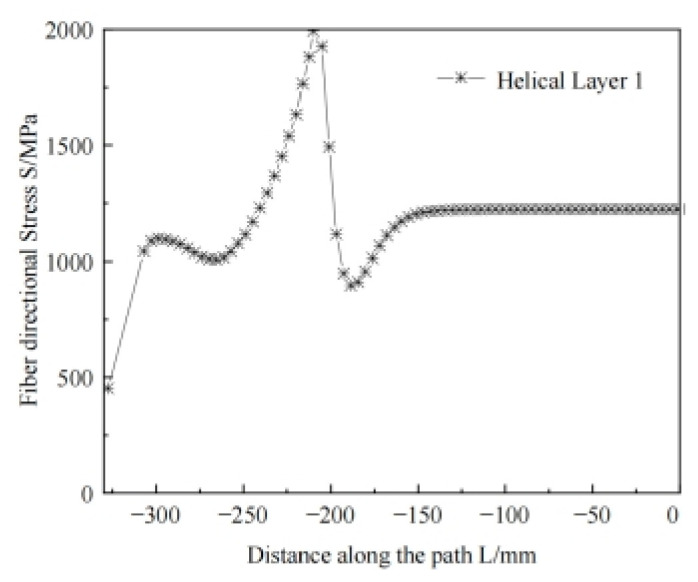
Optimized shell structure dome fiber direction stress [114].

In addition to tension control, geometric discontinuities in dome, conical, or end-turning regions remain a major source of defects in filament-wound structures. Abrupt curvature changes may cause fiber accumulation, gaps, thickness non-uniformity, and local stress concentration. Siegl et al. [113] developed a thermoplastic winding process that combines in situ impregnation with infrared consolidation. By introducing a rotatable consolidation shaft and progressive winding angle control, stable fiber placement was achieved in the end-turning region at a constant linear speed of approximately 470 mm/min, as shown in Figure 24. This strategy improves path realization in geometrically difficult regions, yet the 50 mm working distance of the infrared heating spot restricted the feasible winding angle to ≥ ± 65°, limiting its applicability to smaller winding angles. From a structural design perspective, Zhou et al. [114] established a refined finite element model considering variable winding angle and thickness, with a strain error below 5.9%. By optimizing the reinforcement start radius, layer number, and bandwidth using a neural network surrogate model and NSGA-II, the maximum fiber direction stress in the dome was limited to below 2000 MPa and the structural mass was reduced by 9.3%, as shown in Figure 25. Since ultimate burst tests were not performed, the safety margin under actual burst conditions still needs experimental verification.

**Figure 26:**
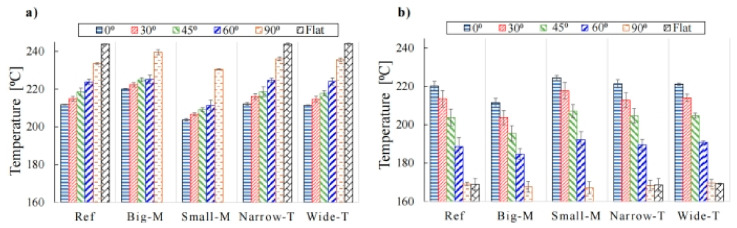
Average temperatures of (**a**) tape and (**b**) substrate and corresponding standard deviations at the nip line for each simulation case as a function of selected θ values [115].

Thermal control becomes particularly important in laser-assisted thermoplastic winding because curved surfaces, varying winding angles, and tape width can produce non-uniform temperature fields. Zaami et al. [115] developed a coupled optical–thermal three-dimensional model to analyze temperature evolution during laser-assisted thermoplastic winding. Their results showed that increasing the winding angle from 0° to 90° decreased the matrix temperature by 40–50 °C, while increasing the tape temperature by 30–40 °C. Smaller mandrel diameters produced matrix temperatures approximately 15 °C higher than those on larger mandrels, and narrow tapes were more prone to lower edge temperatures, which may lead to non-uniform interlayer bonding, as shown in Figure 26. Chang et al. [116] further simulated the moving heat source in Abaqus and found that the temperature peak occurs in the outermost layer, where excessive heating may exceed the resin decomposition limit and cause thermal damage. These modeling studies provide useful guidance for heat source regulation, while their predictive accuracy is still affected by simplified thermo-physical properties, contact assumptions, and boundary conditions. Therefore, thermal modeling should be integrated with experimental calibration rather than used as an isolated prediction tool.

The studies reviewed above show that tension regulation, winding speed control, robotic feedback, end-region path adjustment, and thermal modeling can each improve specific aspects of process stability. Nevertheless, these approaches are still often developed around individual control variables, whereas defects in thermoplastic winding usually arise from the coupled effects of tension, heat input, compaction pressure, tape/roving deformation, mandrel curvature, and cooling history. For example, increasing tension may improve fiber alignment and compaction, but may also increase fiber abrasion or path instability; reducing winding speed may enhance heating and interlayer bonding, but may reduce productivity and promote excessive matrix flow; improving thermal input may strengthen consolidation, but may also introduce local overheating or temperature gradients on curved surfaces. Therefore, the central issue is not to maximize a single parameter, but to identify a stable processing window in which fiber placement accuracy, interlayer bonding, dimensional consistency, and production efficiency can be simultaneously maintained. Future research should therefore focus on integrated process-structure control. Such an approach is essential for improving interlayer bonding consistency, reducing defect accumulation in end regions, and extending thermoplastic filament winding from conventional axisymmetric parts toward more complex load-bearing CFRTP components.

### 3.5. Automated Fiber Placement

Automated fiber placement (AFP) of carbon fiber-reinforced thermoplastic composites is a digital and automated near-net-shape manufacturing process. In this process, a multi-axis motion system drives the placement head to deposit carbon fiber tapes pre-impregnated with thermoplastic resin onto the mold surface under the pressure exerted by a compaction roller. Integrated heat sources, such as laser or infrared heating, locally melt the resin to achieve interlayer bonding with previously placed layers, followed by in situ consolidation during cooling. This process offers high manufacturing quality, a high degree of automation, and significant design flexibility, making it particularly suitable for the rapid fabrication of large primary load-bearing aerospace structures, such as aircraft wings and fuselage skins.

The central difficulty of thermoplastic AFP lies in achieving reliable in situ consolidation within a very short thermal and mechanical interaction time. For semi-crystalline thermoplastics, the tape and substrate must be heated sufficiently to reduce melt viscosity and enable intimate contact, molecular interdiffusion, and crystallization control during subsequent cooling. Insufficient temperature or residence time leaves the matrix too viscous to fill surface asperities and expel entrapped air, leading mainly to interlaminar voids and weak bonding. Existing void control strategies can be broadly divided into two categories: process window optimization and auxiliary consolidation strategies.

**Figure 27:**
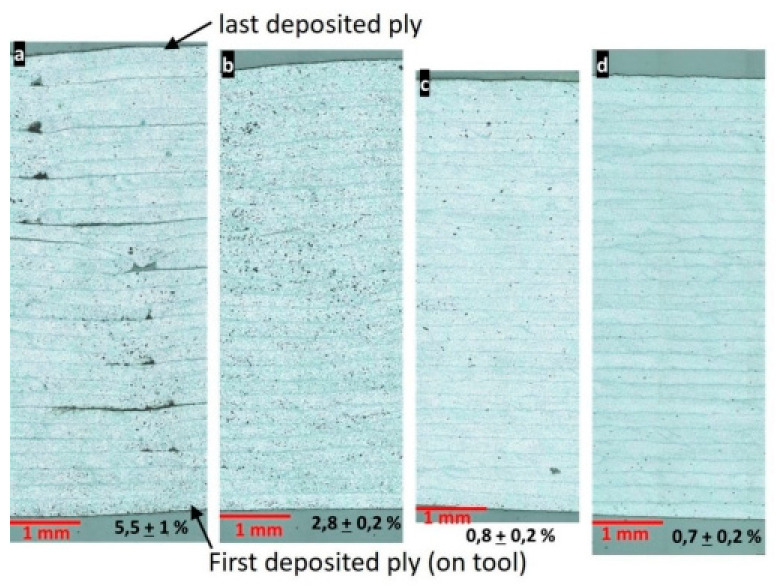
Images of cross-section laminates (**a**) 25/350, (**b**) 250/350, (**c**) 25/450 and (**d**) 250/450 with the associated void content [117].

Researchers often explore suitable processing windows through parameter optimization to reduce porosity. Restif et al. [117] revealed the strong influence of different thermal histories on void formation by varying the laser setpoint temperature and tool temperature. At a relatively low laser setpoint temperature of 350 °C with an unheated tool, both interlaminar and intralaminar voids were observed, with the interlaminar void content reaching approximately 3.12%. When the tool temperature was increased to 250 °C, intimate contact between adjacent layers was improved, and the dominant defects shifted to intralaminar voids, with an intralaminar void content of approximately 2.71%. When the laser setpoint temperature was further increased to 450 °C, the intralaminar void content decreased to approximately 0.8% and 0.7% for the unheated and heated tool conditions, respectively. Figure 27 shows the cross-sectional morphologies of laminates manufactured under different laser and tool temperature conditions. Grimsley et al. [118] further examined the limits of this coupled processing window from an engineering perspective. Their results showed that PEEK laminates achieved good interlayer bonding at a laser temperature of 500 °C, a mold temperature of 120 °C, and a placement speed of 100 mm/s. In contrast, LM-PAEK laminates maintained low porosity even at a higher placement speed of 400 mm/s under conditions of 450 °C laser heating and an 80 °C mold temperature. These findings indicate that parameter optimization is the most direct and industrially accessible route for porosity reduction, but its effectiveness is strongly material-dependent and cannot be directly transferred across different thermoplastic matrices.

**Figure 28:**
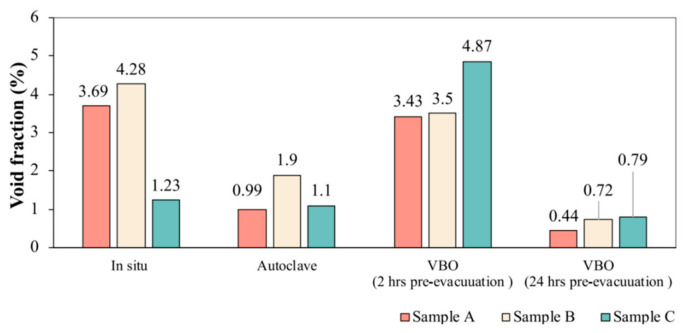
Void content of all fabricated samples, in situ consolidated and post-processed with an autoclave or VBO methods [119].

**Figure 29:**
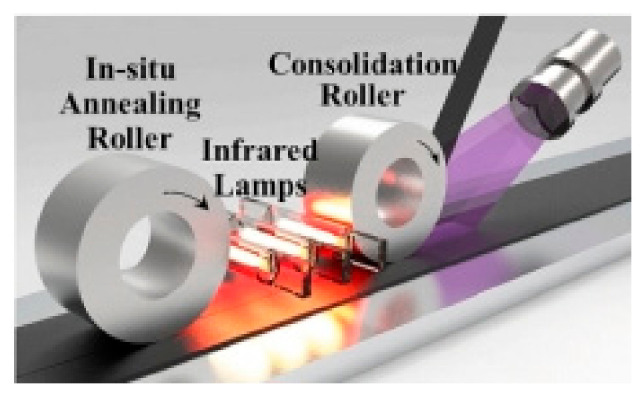
LAFP with the IIA process [120].

The second category of strategies employs novel process routes to reduce porosity. Karimi et al. [119] demonstrated that combining a low-compaction-force AFP process with long-duration vacuum bag only (VBO) post-treatment can replace traditional autoclave processing. As shown in Figure 28, Sample A, fabricated with a compaction force of 300 N and a layup speed of 100 mm/s, exhibited an initial void content of 3.69% but possessed the highest void connectivity. After 24 h of vacuum hold during VBO processing, the void content was significantly reduced to 0.44%, even outperforming autoclave post-processing (0.9%). This approach offers a new pathway for manufacturing high-performance, low-cost aerospace thermoplastic composites; however, the 24 h vacuum hold time considerably extends the production cycle, and its feasibility for high-rate batch production requires further assessment. Wang et al. [120] proposed another strategy that combines laser-assisted AFP with in situ infrared annealing. In this method, infrared heating and a secondary compaction roller are applied immediately after tape placement to induce remelting and controlled cooling, as shown in Figure 29. Without reducing placement efficiency, this approach reduced porosity from 4.87% to 2.05% and increased crystallinity from 18.6% to 33.8%. Meanwhile, the interlaminar shear strength (ILSS) and Mode-I fracture toughness increased by 239.7% and 292.4%, respectively. The resulting improvements are comparable to those achieved by conventional post-placement reconsolidation processes, while avoiding repeated placement operations, demonstrating a favorable balance between performance and manufacturing efficiency. Yet this study only used unidirectional layups, without addressing the multi-angle layups and complex curved geometries typical of actual aerospace structures.

**Figure 30:**
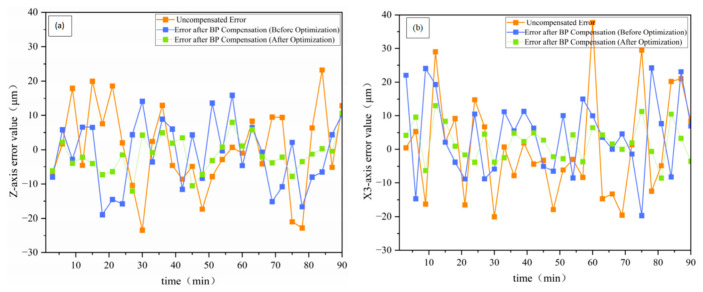
Comparison of errors before and after optimization for the (**a**) Z-axis and (**b**) the X3-axis [121].

Precise trajectory control is essential for ensuring that fibers are placed along the designed orientations and for achieving predictable geometric accuracy and structural performance. Xia et al. [121] developed a ten-axis CNC architecture based on the Siemens 840Dsl system, integrating the Z, X1–X3, SP, C, and V1–V3 axes to enable coordinated control of both automated fiber placement and narrow-tape winding. They further proposed a thermal error compensation model based on a genetic algorithm-optimized BP neural network. The results showed that the system achieved a repeat positioning accuracy of ±1.5 μm, meeting the ±5 μm precision requirement for aerospace composites. After thermal error compensation, the maximum error of the Z-axis decreased from 18.94 μm to 12.01 μm, while that of the X3-axis decreased from 24.22 μm to 11.31 μm. Figure 30 compares the thermal errors of the Z and X3 axes before and after compensation. This work extends the motion capability beyond conventional six-axis systems and improves manufacturing accuracy through intelligent compensation, offering significant engineering value for the fabrication of complex aerospace composite structures. Experiments used only T300 carbon fiber; the method’s applicability to high-modulus fibers and complex curved geometries remain unverified. McArthur et al. [122] extended the functionality of the ITRA toolkit to allow synchronized control of six internal robot axes and six external axes, including a linear track, a rotating mandrel, and material feed rollers, thereby achieving integrated real-time control of an AFP system with a total of twelve degrees of freedom. Experiments were conducted on an AFP unit equipped with a KR120 robotic arm, a KL400 linear track, and an Accudyne single-tow placement head. Path offset commands generated from external servers such as MATLAB were used to dynamically adjust the end-effector position and material feed during processing. The system achieved response delays as low as 60 ms. This work represents one of the first demonstrations of low-latency coordinated control for a multi-degree-of-freedom AFP system, providing an important technical foundation for online defect detection and real-time path correction. Real-time integration of sensor data such as laser profilometers and closed-loop path correction have not yet been implemented.

**Figure 31:**
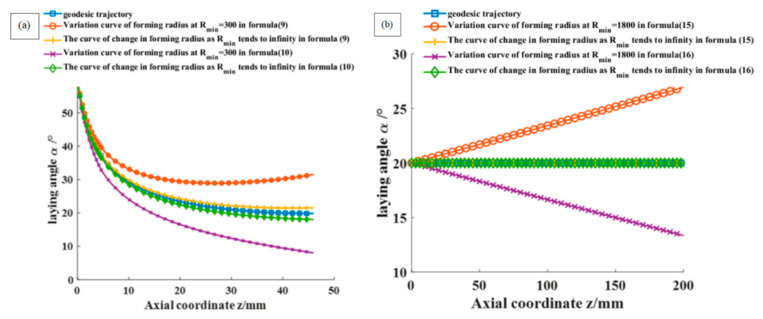
Range of laying angle for the prepreg place placement without wrinkle defects in (**a**) the left ellipsoidal dome section and (**b**) the cylinder section [123].

**Figure 32:**
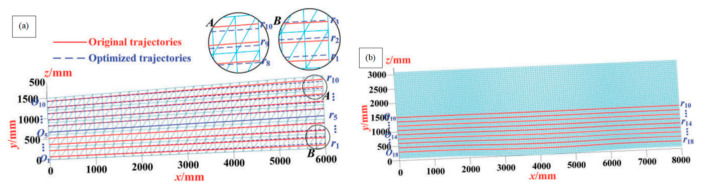
Placement trajectory comparison before and after optimization on (**a**) hyperbolic and (**b**) free-form mesh surfaces [124].

When trajectory planning targets highly complex free-form surfaces, Wang et al. [123] proposed a wrinkle-free path planning method based on differential geometry. The study derived wrinkle-free placement differential equations for both ellipsoidal dome sections and cylindrical sections. For the ellipsoidal dome, under conditions of an initial placement angle of 60°, a prepreg width of W = 6.35 mm, and a minimum forming radius of R_min_ = 300 mm, forward calculation using the wrinkle-free differential equation yielded a feasible terminal placement angle range of [8.03°, 18.04°] ∪ [21.54°, 31.55°] for the left dome. For the cylindrical section, with an initial placement angle of 20° and R_min_ = 1800 mm, the wrinkle-free placement angle range was determined to be [13.35°, 26.94°]. Figure 31 illustrates the wrinkle-free placement angle ranges for both the ellipsoidal dome and cylindrical sections. For the right ellipsoidal dome with a different height from the left dome, a geometric coverage model was first used to generate all feasible paths, after which a wrinkle criterion was applied for validation. The optimal initial placement angle range that avoids wrinkles, gaps, and overlaps was determined to be [60.56°, 90°]. This method enables direct determination of feasible placement regions on surfaces of revolution, significantly improving path planning efficiency and reducing trial-and-error costs, while providing theoretical guidance for the automated manufacturing of composite pressure vessels. Zhang et al. [124] studied trajectory optimization for AFP on triangular mesh surfaces. The STL mesh was first reconstructed into quadratic surface patches using the Nagata interpolation method to recover geometric information and improve path calculation accuracy. A fourth-order Runge–Kutta method in the parametric domain was then used to solve placement paths with specified geodesic curvature on these patches, enabling high-precision trajectory generation on discrete models. Based on this framework, local and global trajectory optimization strategies were proposed. Linear programming simultaneously optimized the initial placement gap and geodesic curvature to limit material deformation while maintaining the gap within 0.2–2.3 mm. Results showed that, with a step size of 0.01 mm, the method achieved trajectory accuracy better than 0.01 mm, even on coarse meshes. Local optimization reduced maximum tow deformation to 89.5–91.5% of the CAD strategy on hyperbolic surfaces and 66.3–86.6% on free-form surfaces (Figure 32a). After introducing additional tows for global optimization, deformation of the most critical tow (P_18_) decreased by 19.6% compared with the CAD strategy and by 7.2% relative to the original global method (Figure 32b), demonstrating an effective approach for high-precision AFP path planning on complex surfaces.

Thermoplastic automated fiber placement provides a promising route for the automated and high-rate manufacture of large CFRTP structures. Its practical application, however, is still limited by a central challenge: consolidation quality and geometric accuracy must be simultaneously controlled within a highly coupled processing system. From the perspective of consolidation quality, parameter optimization remains the most direct approach to reducing porosity. Key variables include laser or infrared power, tool temperature, placement speed, and compaction force, which should be adjusted synergistically rather than independently. Insufficient heat input or pressure duration may restrict interfacial contact and molecular chain interdiffusion, whereas excessive thermal input may induce deconsolidation, non-uniform crystallization, or local degradation. Auxiliary consolidation strategies can further improve laminate quality, but they also introduce new trade-offs in production cycle, equipment complexity, energy consumption, and process transferability. Future development should therefore move beyond empirical parameter selection toward model-assisted and sensor-feedback-driven AFP processes.

### 3.6. Additive Manufacturing

Additive manufacturing (AM), or 3D printing, is a technology that builds three-dimensional structures through layer-by-layer material deposition. The target geometry is first sliced into two-dimensional layers, after which a printing head deposits material along predefined paths to form the final structure [125,126]. Typical AM processes encompass stereolithography (SLA), fused deposition modeling (FDM), selective laser sintering (SLS), selective laser melting (SLM), and laminated object manufacturing (LOM) [127,128,129]. With the rapid progression of AM technologies, the additive manufacturing of carbon fiber-reinforced composites has garnered increasing attention due to its capacity to fabricate intricate structures and components with high specific strength [130,131]. Different reinforcement forms correspond to distinct AM processes: short-fiber composites are typically produced via FDM, SLS, or DLP; fabric-reinforced materials through LOM; and continuous fiber composites predominantly by FDM [132,133]. Owing to its simplicity and cost-effectiveness, FDM has emerged as the most prevalent method for CFRC printing [134,135,136,137].

**Figure 33:**
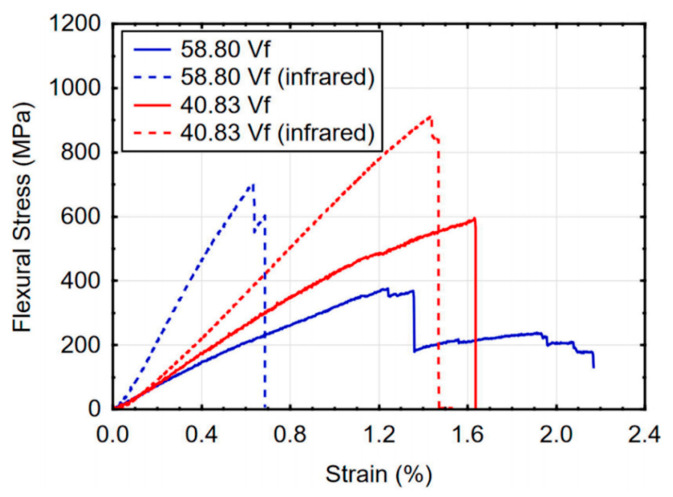
The results of three-point bending tests applied to CFRTP samples: stress–strain curves [138].

**Figure 34:**
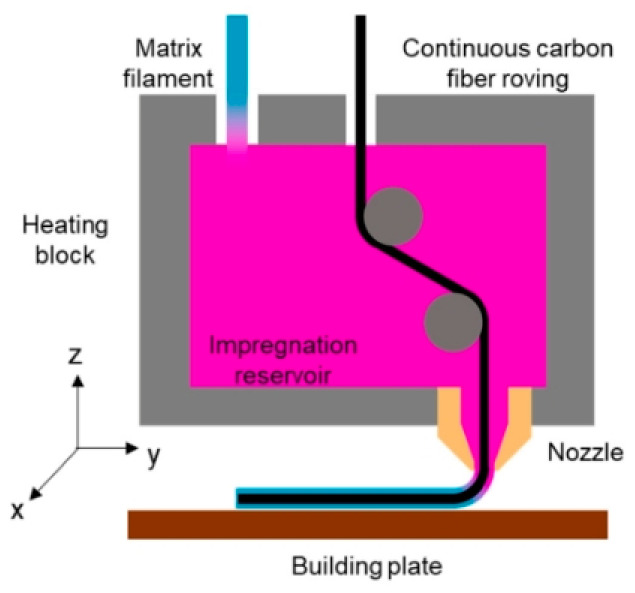
Printhead design featuring pin-assisted fiber spreading and extended impregnation path [139].

For continuous fiber CFRTP additive manufacturing, the main difficulty lies in achieving sufficient melt impregnation and interlayer consolidation within the limited pressure and short residence time of extrusion-based printing. Current studies have addressed this issue mainly through process optimization and ultrasonic assistance. In terms of process optimization, Vatandaş et al. [138] developed a melt impregnation production line integrating fiber spreading, polymer melt impregnation, and forming molds to produce CF/PEEK prepreg filaments with adjustable fiber volume fractions. Infrared heating was further applied to raise the temperature of the deposited layer surface and enhance interlayer fusion. As shown in Figure 33, when the fiber volume fraction reached 58.80%, the specimens achieved a flexural modulus of 134.16 GPa and flexural strength of 721.08 MPa, representing increases of 335.16% and 96.40% compared with the non-heated condition. The highest flexural strength of 900.43 MPa was obtained at a lower fiber volume fraction of 40.83%, indicating that excessive fiber loading can aggravate insufficient wetting and porosity. An et al. [139] proposed an in situ needle-assisted melt impregnation printhead in which a mechanical fiber spreading mechanism was integrated directly inside the nozzle. Multiple fixed needles were arranged within the heating chamber to disperse and spread the fiber bundle, extending the impregnation path of the molten polymer and improving resin infiltration efficiency (Figure 34). Experiments showed that, under optimized conditions (260 °C, 100 mm/min, 0.5 mm layer height), the four-needle configuration achieved an impregnation degree of up to 91.2%, significantly higher than that of conventional needle-free nozzles.

**Figure 35:**
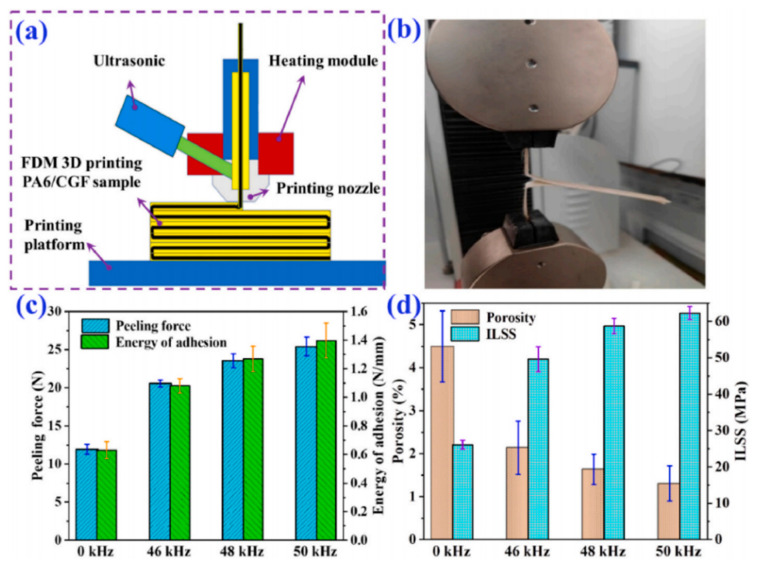
(**a**) Schematic diagram of CGF/PA6 FDM 3D printing process; (**b**) experimental demonstration of interlaminar delamination; (**c**) R between layers, (**d**) porosity and ILSS for 3D printing samples [140].

**Figure 36:**
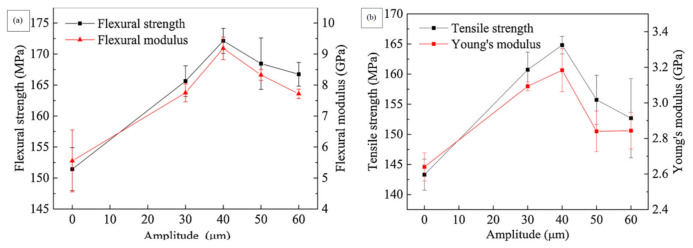
(**a**) Flexural strength and flexural modulus of CF/PLA composites vs. ultrasonic amplitude, and (**b**) tensile strength and Young’s modulus of CF/PLA composites vs. ultrasonic amplitude [141].

Ultrasonic assistance can further improve impregnation. Zhang et al. [140] synchronized an ultrasonic vibration system with the nozzle, directly applying ultrasonic energy to the molten PA6 matrix to enhance interfacial bonding. Compared with conventional FDM, ultrasonic treatment significantly improved fiber wetting and interlayer adhesion: at 50 kHz, porosity decreased from 4.52% to 1.33%, peel force increased from 12.72 N to 25.52 N, and ILSS rose from 26.13 MPa to 62.30 MPa (Figure 35). Correspondingly, tensile strength, flexural strength, flexural modulus, and notched impact strength increased by 54.57%, 28.63%, 41.32%, and 22.54%, respectively. Qiao et al. [141] further enhanced the interface through ultrasonic pretreatment of carbon fibers before printing. Immersing fibers in a PLA solution under ultrasonic cavitation and micro-jet effects produced surface micro-etching, increasing roughness and mechanical interlocking. At an ultrasonic amplitude of 40 μm, tensile and flexural strengths increased by 34% and 29%, respectively, while the flexural modulus improved by 65% (Figure 36). This purely physical approach improves interfacial bonding without risking matrix thermal degradation. Ultrasound-assisted methods tackle the problem at the interfacial level through physical field coupling, achieving significant improvements, yet they involve complex process control and high equipment retrofitting costs.

4D printing extends additive manufacturing by introducing a time dimension, enabling printed structures to undergo programmable changes in shape or properties under external stimuli such as heat, humidity, electric or magnetic fields, and light. For carbon fiber-reinforced thermoplastic composites, this technology not only enables integrated fabrication of complex structures but also improves response speed, controllability, and mechanical performance through continuous carbon fiber reinforcement. Consequently, it shows strong potential in applications such as deployable aerospace structures, soft robotics, and smart medical devices.

**Figure 37:**
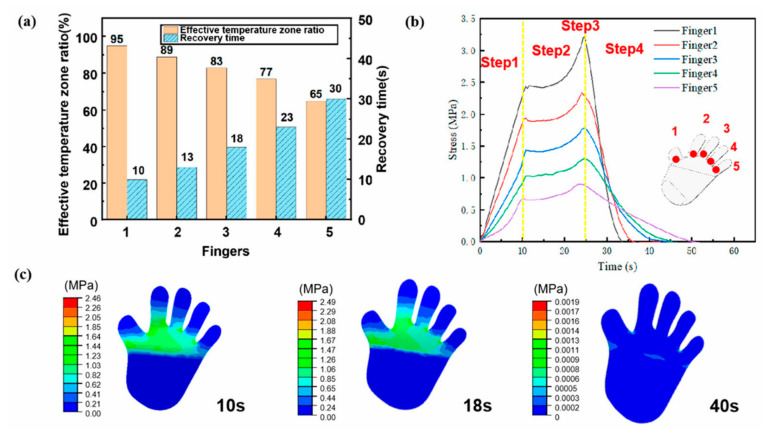
(**a**) Effective temperature zone and recovery time for each finger. (**b**) Stress variation during the electrically driven recovery process. (**c**) Stress distribution at different recovery steps [142].

In terms of programmable fiber paths and structural response, Wang et al. [142] achieved programmable deformation sequencing by regulating the regional distribution of continuous carbon fibers to generate a non-uniform temperature field under electrical stimulation. Two structures were demonstrated: a thickness gradient structure and a hand-shaped gripper. In the gradient structure, varying the PLA thickness from 1 mm to 2.5 mm adjusted the fiber content, producing an axial temperature gradient from 100.5 °C at the thin end to 46.2 °C at the thick end; under 6 V, the structure completed spiral deployment within 18 s. For the hand-shaped structure, different regional fiber volume contents were assigned to the five fingers, ranging from 0.95% to 0.60%, and the simulation results showed sequential recovery within 10–30 s, demonstrating low-voltage programmable deformation (Figure 37). Kumar et al. [143] investigated the influence of continuous carbon fiber orientation and infill density on the bending recovery behavior of PLA-based shape memory composites. Vertical placement yielded the highest recovery ratio reached 92.97%, while horizontal placement produced the best fixation ratio reached 91.78%; an infill density of 40% provided the optimal fixation performance.

**Figure 38:**
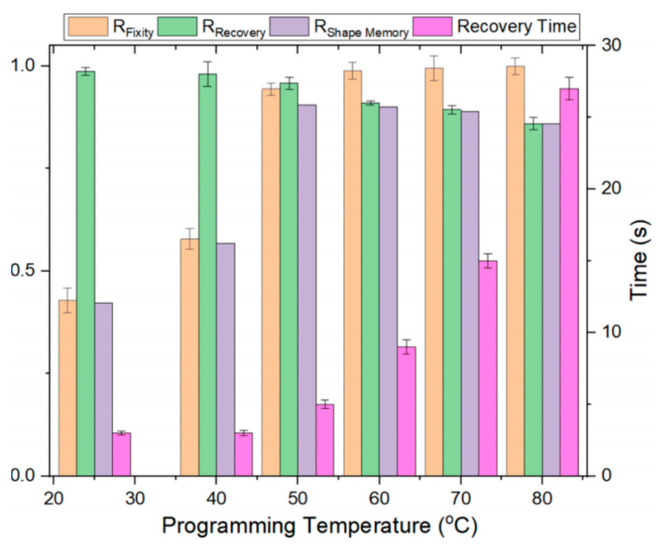
Results of shape memory test of the CCFRP samples. In this test, the programing and recovery were made inside a water bath [144].

**Figure 39:**
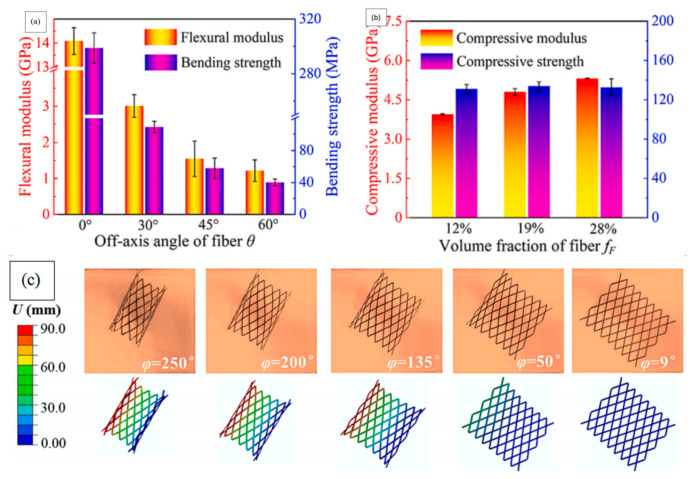
Systematic characterization of co-printed continuous CF/TP composites. (**a**) Statistics of flexural modulus and bending strength; (**b**) modulus and strength under longitudinal compression versus fiber volume fraction f_F_; (**c**) experiments and simulations of the shape recovery process for the mesh structure [145].

From a processing perspective, coaxial extrusion additive manufacturing has become an effective approach for fabricating continuous carbon fiber-reinforced shape memory composites. Fallah et al. [144] fabricated continuous carbon fiber-reinforced shape memory polymers via coaxial 4D printing, achieving increases of 276% in tensile modulus and 246% in flexural modulus compared with pure PLA. Programming temperature strongly affected shape-memory behavior: low-temperature programming (25–50 °C) produced rapid recovery with >95% recovery ratio but weaker fixation, whereas programming near or above Tg resulted in nearly complete fixation but slower and less complete recovery (Figure 38). Zeng et al. [145] further characterized 4D-printed continuous carbon fiber-reinforced shape memory polymer composites fabricated through a fiber/polymer in-situ impregnation co-extrusion process using micro-CT, mechanical testing, SEM fracture analysis, and finite element simulation. The composites exhibited pronounced anisotropy: increasing the fiber off-axis angle from 0° to 30° reduced the tensile modulus by 88.2% and the bending strength by 63.6%, whereas increasing the fiber volume fraction from 12% to 28% gradually improved the longitudinal compressive modulus (Figure 39a,b). Nevertheless, both the rectangular specimen and the mesh structure showed shape recovery ratios above 96%, and the experimental recovery process of the mesh structure agreed well with the finite element simulation (Figure 39c). This study establishes a systematic understanding linking fiber architecture, internal defect distribution, load-bearing behavior, and shape-memory performance in CF/thermoplastic 4D-printed composites, supporting their application in load-bearing and controllable morphing structures.

For CFRTP additive manufacturing, the major challenge is not simply the incorporation of carbon fibers into printable thermoplastic matrices, but the stable fabrication of parts with predictable mechanical performance. Continuous fiber printing and high-temperature matrices such as PEEK can significantly improve stiffness and strength, but they also increase the difficulty of melt impregnation, nozzle deposition, interlayer fusion, and thermal history control. In situ heating, ultrasonic assistance, and pre-impregnated feedstocks have improved interfacial bonding and reduced defects, yet these methods often introduce additional equipment complexity and may reduce printing efficiency or geometric accuracy. More importantly, many reported improvements are still validated using small specimens, selected fiber paths, or specific loading directions, while the behavior of large, thick, multidirectional, or structurally complex components remains less certain. Therefore, future development should place greater emphasis on process repeatability, interlayer quality control, fiber path design for load-bearing applications, and standardized evaluation methods, so that CFRTP additive manufacturing can move from functional prototyping toward reliable structural manufacturing.

### 3.7. Comparative Assessment of Forming Processes

To provide a more direct comparison, Table 3 summarizes the applicable forms, maturity levels, major advantages, and key limitations of stamping, injection, pultrusion, filament winding, AFP, and additive manufacturing.

Although CFRTP exhibit advantages such as high specific strength, weldability, and recyclability, their adoption remains uneven across different industrial sectors. The gap between technical potential and broad commercialization is mainly associated with material cost, equipment investment, forming cycle, quality control requirements, and process scalability. As shown in Table 3, mature processes such as stamping and injection molding rely on well-established industrial equipment and short forming cycles, making them more suitable for high-volume production. Pultrusion and filament winding provide scalable manufacturing routes for continuous profiles and axisymmetric structures, respectively, but their economic advantages depend largely on geometric regularity and stable process control. AM offers a high degree of geometric design freedom, but its further development is still limited by low forming efficiency, restricted part size, and insufficient interlayer bonding strength. In contrast, AFP has a high level of automation and can enable rapid placement, flexible design, and good forming quality; nevertheless, high equipment investment and the high cost of quality thermoplastic prepreg tapes remain important factors limiting its broader application. Therefore, the selection of CFRTP forming technologies should not be guided solely by performance, but should be comprehensively evaluated according to the specific application scenario, production scale, and cost acceptability.

To further clarify the process–structure–property relationship in CFRTP forming, Table 4 summarizes the general effects of common forming parameters on processing-induced structural features and the resulting mechanical performance. These relationships should be understood as qualitative trends within an appropriate processing window, rather than universal linear rules.

Taken together, Table 4 indicates that the mechanical performance of CFRTP is not determined by processing parameters alone, but by the structural quality produced under a given processing window. Adequate heating, pressure and consolidation time can improve resin flow, fiber impregnation, void reduction and interlayer contact, but excessive thermal or mechanical input may cause resin loss, degradation, residual stress or non-uniform crystallization, thereby offsetting the benefits of consolidation. For semi-crystalline matrices, the cooling history is also important because the resulting crystallinity and crystalline morphology affect the stiffness–toughness balance as well as interlaminar and compressive performance. In processes involving large deformation or strong shear, changes in fiber orientation, fiber length, tow movement, wrinkling and thickness distribution may disturb the intended load-bearing path, leading to anisotropic or geometry-dependent mechanical responses even when the same material system is used. The interface further links the formed microstructure with macroscopic performance, because insufficient wetting or poor interfacial compatibility promotes fiber pull-out, interfacial debonding and delamination, whereas suitable sizing or interfacial modification improves IFSS, ILSS, flexural strength and delamination resistance. Therefore, process optimization should be judged not only by nominal parameters such as temperature, pressure, speed and time, but also by whether the final structure achieves sufficient impregnation, low void content, controlled crystallinity, stable fiber architecture and reliable interfacial bonding.

To further connect manufacturing with property prediction, predictive modeling should treat processing-induced structural features as intermediate variables between forming conditions and structural performance. Conventional micromechanical models can estimate ply-level properties from constituent properties, fiber volume fraction, and interfacial load transfer, whereas multiscale models can further transfer micro- or meso-scale information to laminate or component-level responses [150,151]. For CFRTP manufacturing, these models should not rely only on ideal material properties, but should incorporate process-dependent features such as void content, impregnation quality, crystallinity, fiber orientation, tow waviness, residual stress, and interfacial bonding. In this way, process simulations and experimental characterization can provide realistic inputs for predicting stiffness, strength, interlaminar failure, dimensional stability, and damage tolerance of final CFRTP structures. However, the accuracy of such predictions still depends strongly on reliable defect quantification, validated constitutive relationships, and structural-scale experimental verification, especially for large, thick, multidirectional, or geometrically complex CFRTP components.

Although process optimization can improve resin flow, impregnation and interlayer bonding within a suitable processing window, it cannot fully eliminate the material-derived limitations associated with high matrix viscosity and insufficient fiber–matrix compatibility. Therefore, the following section further discusses interface engineering as a complementary strategy for improving wetting, shortening effective impregnation pathways, and enhancing load transfer in CFRTP systems.

## 4. Interfacial Regulation

The interface, acting as the transitional zone between the fiber and matrix, assumes a pivotal role in stress transfer. Robust interfacial adhesion facilitates efficient load transmission and optimizes the performance of both components. Nevertheless, the intrinsic inertness of carbon fiber surfaces and thermoplastic matrices, coupled with the high melt viscosity of thermoplastics, impedes effective impregnation during processing. Although process optimization can enhance macroscopic mold filling, resin flow within the confined spaces of fiber bundles remains constrained, resulting in inadequate wetting and impregnation. Furthermore, excessively severe processing conditions may provoke thermal degradation and interfacial instability, ultimately compromising composite performance.

Therefore, mitigating viscosity-induced constraints through interfacial regulation align more closely with the inherent nature of thermoplastic systems and constitute a more viable pathway for enhancing processing quality. Rather than modifying bulk rheology, interfacial regulation centers on customizing the boundary conditions that govern resin flow, wetting, and diffusion. These strategies can be broadly classified into three categories: (i) enhancing initial wetting through interfacial modification, (ii) reducing infiltration and diffusion pathways via structural design, and (iii) functional interlayer design, which improves local resin supply and interlaminar toughness by introducing resin-rich or toughening phases into the interlaminar region [23,152,153,154]. This section provides an overview of recent representative approaches aimed at improving the flow and impregnation of high-viscosity thermoplastic resins through interfacial regulation.

### 4.1. Interfacial Chemical Modification: Improving Wettability

**Figure 40:**
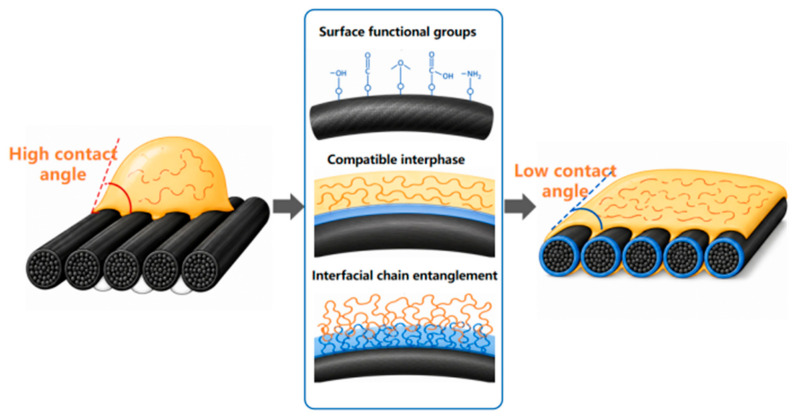
Mechanism of surface functionalization for enhancing initial wetting and interfacial compatibility in CFRTP.

Among the various interfacial regulation strategies, interfacial modification stands out as the most versatile approach for enhancing the wetting and impregnation behavior of high-viscosity thermoplastic resins, as it directly addresses the fiber/resin interface. Sizing agents, typically in the form of polymer-based solutions or emulsions, can be applied to the carbon fiber surface through dipping or spraying to create a thin polymer coating, thereby improving fiber wettability. By designing functionalized sizing agents with high compatibility with the thermoplastic matrix, the effective wetting resistance at the fiber/resin interface can be substantially reduced without altering the inherently high viscosity of the resin. This promotes resin spreading, infiltration, and interfacial bonding. The underlying mechanism involves increasing fiber surface energy, introducing chemically compatible or reactive functional groups, and constructing a stable interfacial transition layer, which collectively provide sufficient driving force for the wetting of high-viscosity melts [155,156,157,158], as illustrated in Figure 40. This section explores various sizing modification strategies.

**Figure 41:**
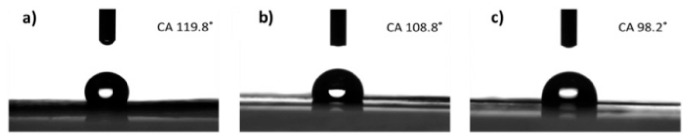
CA results for neat (**a**) as well as sized CF with PEI (**b**) and PEI-PEEK blend (**c**) [159].

**Figure 42:**
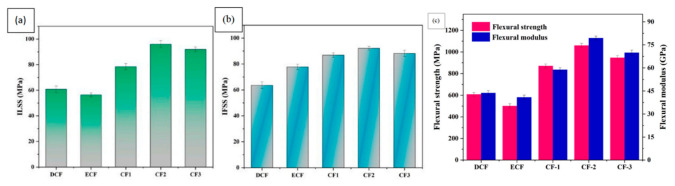
The (**a**) ILSS, (**b**) IFSS and (**c**) flexural properties of CF/PEEK composites [160].

Homologous or chemically similar thermoplastic sizing agents can form a highly continuous and compatible interphase, and are therefore regarded as one of the most direct and effective approaches for reducing the equivalent impregnation resistance in high-viscosity systems. Yavuz et al. [159] developed PEI and PEI-PEEK blended thermoplastic sizing agents, achieving a synergistic regulation of fiber surface energy and morphology. Specifically, the PEI-PEEK sizing increased the surface free energy of carbon fibers from 5.67 mJ/m^2^ to 13.13 mJ/m^2^ (an increase of ~131%), while reducing the contact angle from 119.8° to 98.2°, as evidenced by the contact angle measurements shown in Figure 41. Meanwhile, a continuous coating layer was formed on the fiber surface, decreasing the surface roughness (Ra) from 3.70 nm to 1.71 nm, thereby providing a more uniform physical interface for the spreading and impregnation of molten PEEK. These improvements in interfacial wettability were directly reflected in macroscopic properties, with the elastic modulus increasing from 14 GPa to 24 GPa and the tensile strength from 558 MPa to 611 MPa. These results indicate that optimizing the physical interface can effectively reduce the equivalent wetting resistance of high-viscosity melts. Yan et al. [160] developed a novel thermoplastic water-soluble sizing agent based on sulfonated PEEK containing cardo groups (S-PEEK-WC). The results showed that, for CF/PEEK composites treated with 1 wt% S-PEEK-WC, the ILSS, flexural strength, and IFSS reached 96.0 MPa (+57.9%), 1060.0 MPa (+56.3%), and 92.1 MPa (+44.8%), respectively, as illustrated by the interfacial performance enhancement presented in Figure 42. The effectiveness of this strategy remains sensitive to sizing chemistry and coating amount: solvent-based systems may raise environmental and processing concerns, while excessive water-soluble sizing can cause surface agglomeration and fiber bundle dispersion problems, ultimately reducing interfacial performance.

**Figure 43:**
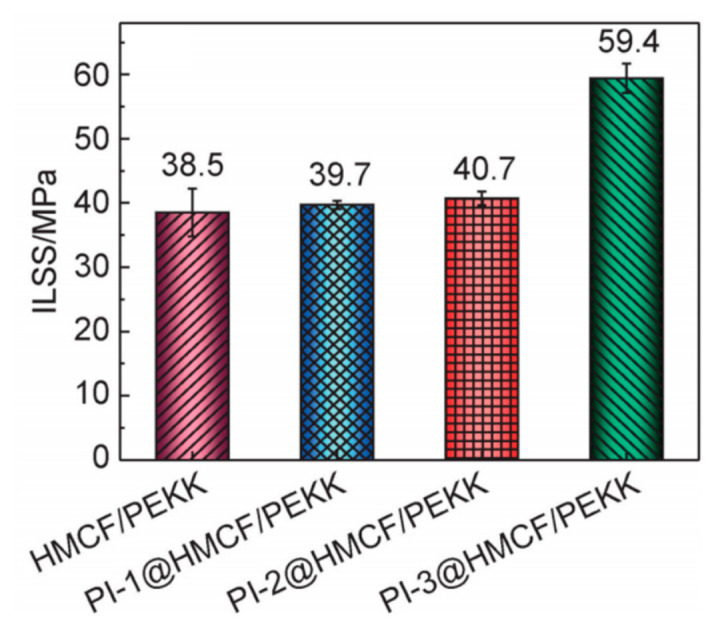
ILSS of modified carbon fibers and the corresponding composites [161].

**Figure 44:**
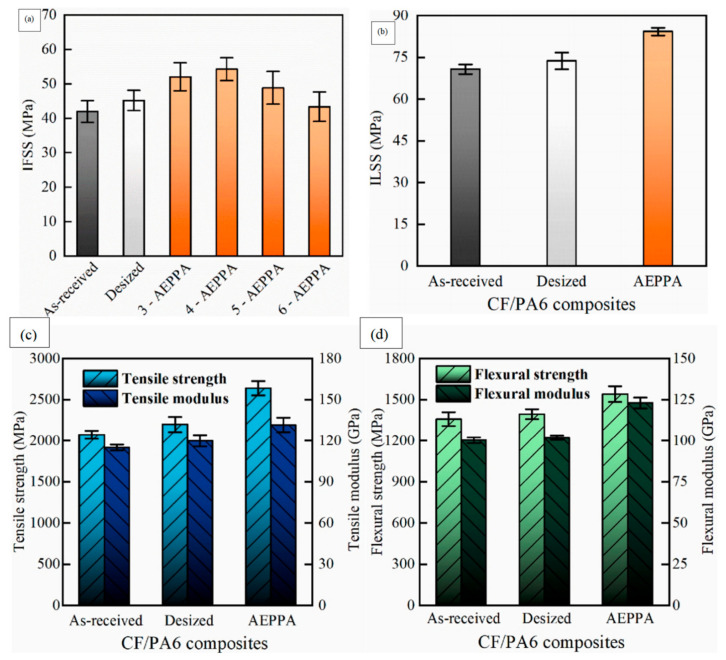
Interfacial and mechanical properties of CF/PA6 under different treatment conditions (**a**) IFSS; (**b**) ILSS; (**c**) tensile properties; (**d**) flexural properties [163].

Heterogeneous sizing agents with high compatibility are also widely employed, as they maintain good wettability while offering greater processing flexibility. Wang et al. [161] synthesized water-soluble thermoplastic polyimide sizing agents for high-strength and high-modulus CF/PEKK composites. The optimized PI-3 sizing increased the O/C ratio on the fiber surface from 0.078 to 0.183 and smoothed the longitudinal grooves of the carbon fibers, indicating improved surface activity and coating coverage. As a result, the ILSS of HMCF/PEKK composites increased from 38.5 to 59.4 MPa, corresponding to an improvement of 54.3%, as shown in Figure 43. Huang et al. [162] designed a water-soluble polyamide acid sizing agent containing reactive alkyne groups. At a sizing concentration of 3 wt%, the water contact angle decreased from 73.92° to 60.65°, while the surface free energy increased from 31.37 to 41.21 mN/m. The IFSS consequently increased from 82.08 to 108.62 MPa, and an interphase of approximately 85 nm was formed, which helped reduce the modulus mismatch between carbon fiber and polyimide resin. Yan et al. [163] designed a water-based aminoethylpiperazine-polyamide (AEPPA) sizing agent, whose molecular structure contains amide groups and piperazine rings similar to those of PA6. With 4 wt% AEPPA sizing, the IFSS of CF/PA6 composites reached 54.2 MPa, representing increases of 20.2% and 29.3% compared to desized and as-received fibers, respectively. Meanwhile, the ILSS, tensile strength, and flexural strength increased by 14.2%, 20.0%, and 10.5%, respectively, as illustrated by the performance comparison under different treatment conditions in Figure 44. These studies suggest that matrix-compatible waterborne sizing agents can improve wetting, chain interdiffusion, hydrogen bonding, and stress transfer across the interface. Their effectiveness, nevertheless, depends strongly on coating concentration and layer uniformity. Insufficient sizing may provide limited interfacial activation, whereas excessive sizing can cause fiber adhesion, surface agglomeration, or a mechanically weak boundary layer, ultimately reducing interfacial performance.

**Figure 45:**
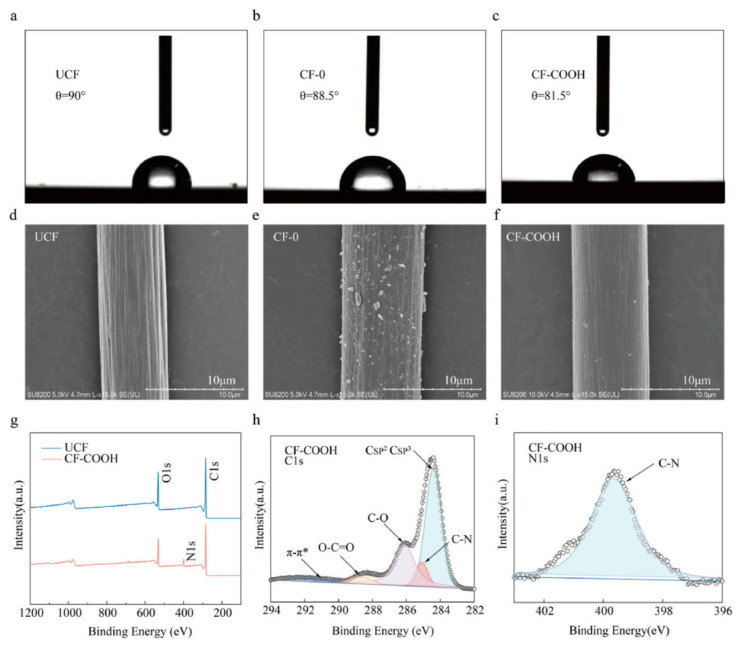
CF surface changes before and after sizing. (**a**–**c**) Dynamic water contact angle, (**d**–**f**) SEM, (**g**–**i**) XPS structural characterization [164].

Functional bridging-type sizing agents compensate for insufficient intrinsic compatibility by introducing highly reactive functional groups. Bao et al. [164] developed an environmentally friendly water-based sizing agent (HPPAEN-COOH), in which abundant carboxyl groups were introduced via hydrolysis. This significantly enhanced the hydrophilicity of the fibers, reducing the contact angle between modified carbon fibers (CF-COOH) and water from 90° to 81.5°, thereby facilitating physical wetting by the resin. Meanwhile, the rich carboxyl groups in the sizing molecules enabled hydrogen bonding and π-π interactions with the fiber surface, forming a stable “molecular bridge”. As a result, the interfacial interaction energy decreased by ~9.0%, accompanied by increases of 25% and 11.3% in storage modulus and flexural strength, respectively. A comprehensive characterization of the fiber surface before and after modification is presented in Figure 45. Qiu et al. [165] designed a sulfonated waterborne epoxy sizing agent with high epoxy functionality, featuring epoxy groups at both ends and a hydrophilic segment in the middle. The highly reactive terminal epoxy groups can undergo covalent crosslinking with the epoxy matrix during curing, forming strong chemical bonding at the interface. Consequently, the interlaminar shear strength increased from 46.52 MPa to 51.74 MPa. The study also highlighted that the hydrophilicity of the sizing agent must be carefully balanced: insufficient hydrophilicity leads to large emulsion particle sizes and poor stability, resulting in non-uniform coatings, whereas excessive hydrophilicity reduces the density of epoxy functional groups per unit area due to elongated molecular chains, thereby weakening interfacial crosslinking. Although this work focuses on thermosetting systems, it provides valuable insights into the balance required in molecular design, which is broadly applicable to functional sizing agents.

However, the effectiveness of sizing agents is strongly dependent on their chemical compatibility with the target thermoplastic matrix and their thermal stability during high-temperature processing. Conventional epoxy-based sizing designed for thermoset composites may degrade or become incompatible with high-performance thermoplastic matrices, leading to interfacial defects or weak boundary layers. In addition, the thickness and uniformity of the sizing layer must be carefully controlled. An insufficient sizing layer may fail to improve wetting, whereas excessive sizing may hinder direct load transfer between the carbon fiber and matrix. Moreover, overly strong interfacial adhesion is not always beneficial, as it may suppress interfacial debonding and crack deflection, thereby reducing damage tolerance. Therefore, sizing design should balance wetting improvement, interfacial adhesion, thermal stability, and toughness.

### 4.2. Interfacial Structural Design: Shortening Infiltration and Diffusion Paths

**Figure 46:**
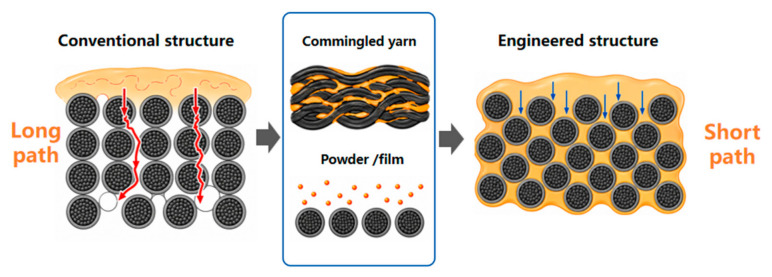
Interfacial structural design for shortening resin impregnation and diffusion pathways in CFRTP.

In addition to interfacial wettability, the characteristic length of resin infiltration and diffusion during processing significantly influences impregnation quality, especially in high-viscosity systems. In conventional continuous fiber bundles and laminated structures, the resin must traverse relatively long distances to achieve complete infiltration, often resulting in inadequate impregnation under high-viscosity conditions. To overcome this limitation, interfacial structural design has been proposed to redistribute the resin at the microscale and shorten the effective diffusion pathways, thereby alleviating viscosity-induced constraints [166,167], such Figure 46. This section focuses on two representative approaches—commingled yarns and powder impregnation—which enhance impregnation by reducing infiltration path length.

Commingled yarns represent one of the most typical strategies in interfacial structural design. In this method, reinforcing fibers and thermoplastic resin fibers are intimately mixed at the fiber bundle scale, enabling a uniform distribution of resin within the bundle prior to processing. Upon heating, the resin fibers melt, and impregnation no longer depends on macroscopic infiltration from the exterior of the bundle. Instead, wetting is achieved through short-range diffusion between adjacent fibers. This transition from “external macroscopic flow” to “intra-bundle microscale diffusion” significantly reduces the characteristic transport length, which is particularly beneficial for high-viscosity systems [80,168,169,170].

**Figure 47:**
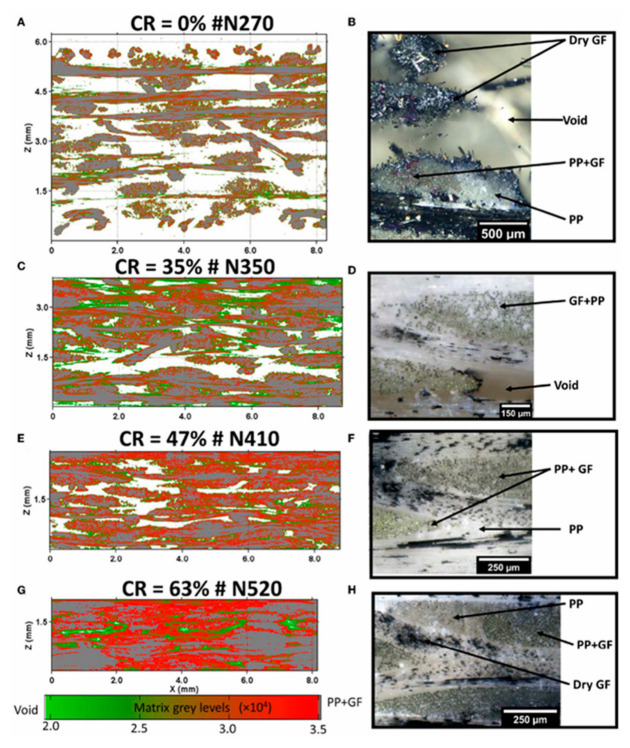
(**A**,**C**,**E**,**G**) Color-coded matrix voxels from µCT 3D images which were segmented using the WCT method using the same scale bar. (**B**,**D**,**F**,**H**) Optical microscopy observations in the XZ plane of the composites samples [172].

Hamada et al. [171] fabricated unidirectional composites using film stacking (FS), unmixed yarns (UYs), and commingled yarns (CYs). Microscopic observations revealed that impregnation is strongly governed by fiber bundle size and intra-bundle porosity. Due to the uniform mixing of reinforcement and matrix fibers, CY not only reduces the size of fiber-rich regions to be impregnated but also increases intra-bundle porosity, resulting in significantly improved impregnation compared to UY and FS. Ono et al. [80] fabricated woven laminates from spread commingled carbon fiber/nylon yarns and reported that the nylon matrix could impregnate the carbon fiber bundle in less than 1 min. Their impregnation model showed that, when the distance from the resin flow front to the center of the fiber bundle was reduced to 10 μm in commingled yarns, complete impregnation could be achieved within approximately 20 s under 1 MPa. By comparison, when the characteristic distance was assumed to be 100 μm, as in a film stacking configuration, complete impregnation required about 3 min and 20 s, even under 10 MPa. Cross-sectional observations further showed no obvious voids in laminates molded at 5 MPa for 5 min, confirming that spread commingled yarn fabrics can achieve rapid impregnation under relatively mild molding conditions. Longer molding times did not significantly improve the flexural properties and even slightly reduced tensile strength and fracture strain, suggesting that excessive thermal exposure may degrade the nylon matrix once impregnation is already complete. Ayadi et al. [172] investigated the impregnation mechanism and fiber bed deformation behavior of biaxial weft-knitted fabrics made from glass fiber/polypropylene commingled yarns using μCT combined with a staged curing method. The CT results showed that, at low compaction levels, the resin was already discretely and uniformly distributed within the fiber bundles, requiring only micrometer-scale diffusion distances after melting to achieve full impregnation. When the compaction ratio exceeded ~60%, the dominant process shifted to resin redistribution between bundles rather than long-range infiltration from the exterior. A comparison of μCT characterization and optical microscopy of resin distribution under different compaction ratios is presented in Figure 47. This study directly visualizes the three-dimensional evolution of resin impregnation in commingled yarns, providing strong experimental evidence for the effectiveness of this approach in shortening infiltration and diffusion paths.

In contrast to commingled yarns, where resin is pre-dispersed within fiber bundles, powder impregnation reduces the characteristic diffusion length to the fiber interface by constructing discretely distributed resin sources on the fiber surface. In this approach, thermoplastic resin is uniformly deposited or attached onto the fiber surface in the form of powders or microparticles. During heating, these particles undergo localized melting, forming multiple independent wetting initiation sites on the fiber surface. Compared with the conventional mode in which a continuous melt infiltrates fiber regions from interlaminar spaces or the bundle exterior, this “multi-point” melting-diffusion mechanism significantly reduces the diffusion distance required for each impregnation event. As a result, high-viscosity resins can achieve effective wetting and coverage of fibers within a limited processing time window. Depending on the powder deposition method, this technique can be broadly classified into wet powder impregnation and dry powder impregnation [173,174,175,176].

**Figure 48:**
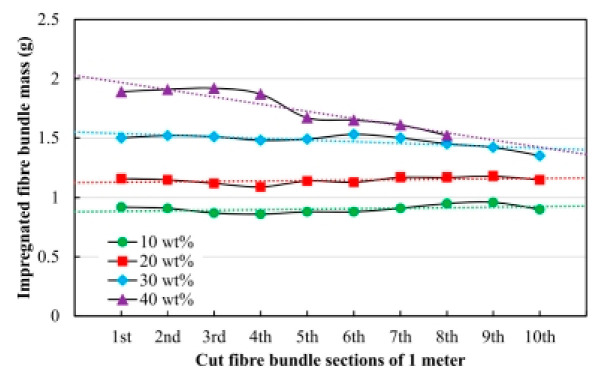
Consistency checks of the impregnation process by resin pick-up test [173].

Wet powder impregnation typically involves dispersing thermoplastic resin powders uniformly in a liquid medium to form a suspension, followed by fiber impregnation through particle adsorption, and subsequent drying and melting to produce prepregs [174,176]. Moradi et al. [173] prepared aqueous slurries containing S2 glass fibers and PAEK resin powders with mass fractions of 10%, 20%, 30%, and 40%, and conducted impregnation experiments using a custom drum-winding setup. The results showed that optimal impregnation was achieved at 10% and 20% concentrations, where resin pickup remained stable with a low standard deviation of 0.03. The effect of slurry concentration on the consistency of resin pickup is illustrated in Figure 48. At higher concentrations, however, the significantly increased viscosity led to particle agglomeration on the roller surface, resulting in elevated fiber tension and increased filament breakage, which ultimately hindered uniform and stable impregnation. Vodermayer et al. [177] established a geometric model of the powder impregnation process to predict the optimal powder particle size and suspension concentration. The results indicated that, for carbon fibers with a diameter of approximately 7 μm and a target fiber volume fraction of 60%, the optimal resin particle size is around 5 μm. Under these conditions, the flow and diffusion distances during melting are minimized, thereby reducing the tendency for void formation. However, excessively fine powders increase suspension viscosity and promote particle agglomeration. Therefore, a practical compromise is to use powders with particle sizes in the range of 15–20 μm.

Dry powder impregnation is conventionally performed within a fluidized bed containing thermoplastic resin powders, wherein particles are electrostatically deposited onto the fiber surface and subsequently melted to fabricate prepregs [173,175,176,178]. Goud et al. [179] documented that airflow velocity must be meticulously regulated during the processing. Excessive airflow can dislodge resin particles that have already adhered to the fiber surface and may also induce particle accumulation at the corona tip, thereby compromising particle charging efficiency and resulting in a diminished resin content in the final prepreg. Ji et al. [180] explored the impacts of airflow pressure, electrostatic voltage, and impregnation time on powder deposition using glass and carbon fibers with PA and PA6 powders. The findings revealed that the quantity of deposited resin initially escalates and subsequently stabilizes with increases in both impregnation time and airflow pressure. Although electrostatic voltage exerts a limited influence on the total deposition amount, it significantly affects the uniformity of particle distribution and adhesion strength on the fiber surface. Furthermore, smaller powder particles demonstrate superior deposition efficiency. Owing to the presence of surface sizing and inferior dispersion, carbon fibers exhibit a lower powder deposition amount compared to glass fibers.

Although interfacial structure design can shorten the resin penetration and diffusion path, its effectiveness relies heavily on the uniform pre-distribution of the thermoplastic phase within or near the fiber architecture. Non-uniform powder deposition, uneven fiber spreading, or unstable commingling may generate resin-rich and resin-poor regions, resulting in local dry spots, voids, or inconsistent interfacial quality. In addition, structural operations such as tow spreading, fiber commingling, and powder impregnation may disturb the original reinforcement architecture, causing fiber misalignment, tow distortion, filament breakage, or reduced textile stability. These issues become more pronounced during complex forming processes, where shear deformation, wrinkling, and local thickness variations may occur. Therefore, this strategy requires precise control of fiber architecture, resin distribution, tension, pressure, and consolidation conditions.

### 4.3. Functional Interlayer Design: Regulating Local Resin Supply and Interlaminar Toughening

Functional interlayer design focuses on the weak interlaminar region of CFRTP laminates, where insufficient resin contact and residual voids can reduce bonding quality and promote delamination. By placing an additional resin-rich or toughening phase between adjacent plies or on prepreg surfaces, the interlayer can act in two stages. During consolidation, it melts, softens, or redistributes locally to improve resin–resin contact and fill interlaminar voids. After solidification, it becomes a toughened layer that changes the way cracks propagate through the laminate. Instead of advancing along a weak and relatively flat interface, the crack is forced to pass through a more deformable or phase-structured region, where plastic deformation, crack deflection, fiber bridging, pull-out, and mechanical interlocking consume additional fracture energy. Therefore, the effectiveness of interlayer design depends on whether the introduced phase can be uniformly distributed, well-bonded to the surrounding matrix, and controlled in thickness and morphology during consolidation [181,182,183].

**Figure 49:**
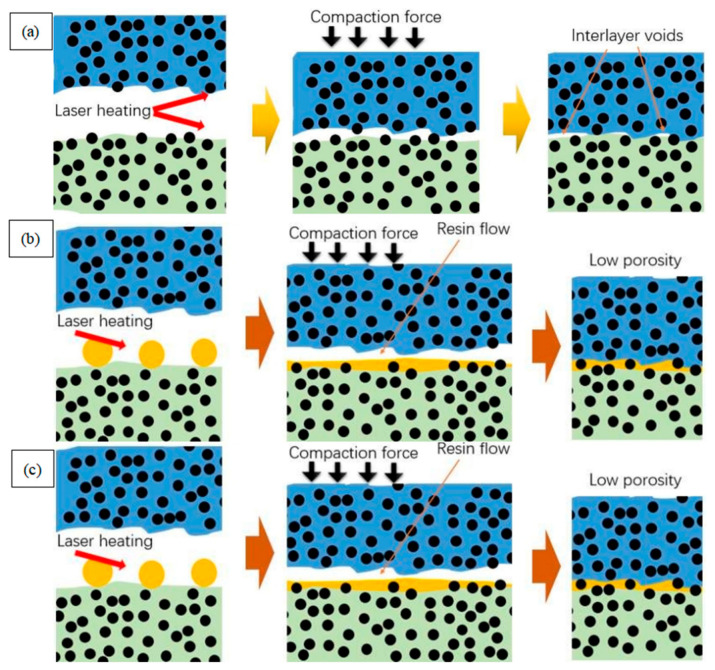
Schematic diagram of resin/fiber distribution: (**a**) during the LAFP process; (**b**) with coated PEEK powder; (**c**) excessive PEEK powder [18].

For CFRTP manufacturing, resin supply interlayers are directly related to processing defects caused by insufficient local resin contact. Wang et al. [18] proposed electrostatically spraying fine PEEK powder onto CF/PEEK prepreg surfaces before laser-assisted fiber placement. During LAFP, the bonding time between adjacent layers is extremely short, and exposed dry fibers on the prepreg surface hinder intimate resin contact. The added PEEK powder, with an average particle size of approximately 20 μm, can rapidly melt under laser heating and fill interlaminar voids. As a result, the porosity of the resin-interleaved laminate decreased to 3.7%, while the resin content increased by only 4.5%; the wedge peel strength increased by 30.1% without repass treatment. The mechanism by which powders flow and fill interlaminar voids is illustrated in Figure 49. This indicates that powder interleafing can locally compensate for insufficient resin supply and improve interlayer bonding within the original processing window. Excessive powder addition, by contrast, may lead to incomplete melting because carbon fibers preferentially absorb laser energy, resulting in residual defects and reduced bonding quality. Therefore, the key issue for powder-type interlayers is to balance coating amount, particle melting, and interlayer resin flow.

**Figure 50:**
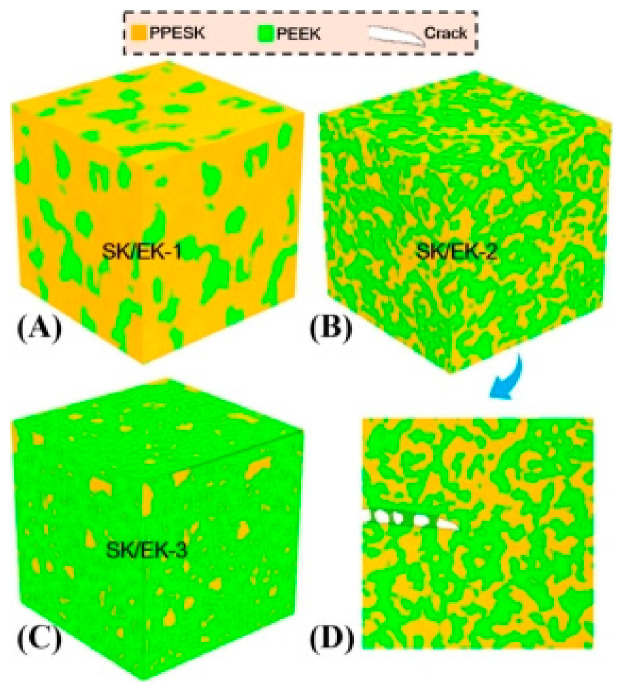
Stereograms of the interlayer phase structures of different CFPWF/PPESK-PEEK composites and the mechanism of action of the bicontinuous phase structure: (**A**) PPESK:PEEK = 4:1. (**B**) PPESK:PEEK = 1:1. (**C**) PPESK:PEEK = 1:4. (**D**) The mechanism of action of the bicontinuous phase structure [184].

Beyond local resin supplementation, phase-structured interlayers can further regulate load transfer and energy dissipation. Li et al. [184] fabricated PPESK/PEEK composite interlayers via an interlaminar spraying technique, with PPESK-to-PEEK ratios of 4:1, 1:1, 1:4, and pure components. The results indicated that the 1:1 ratio produced a uniform bicontinuous phase structure, leading to optimal overall mechanical performance. The tensile strength reached 477 MPa, representing increases of 30.79% and 8.82% compared to pure PPESK and pure PEEK interlayers, respectively, while the flexural strength reached 338 MPa, with corresponding improvements of 25.90% and 112.50%. Microscopic observations confirmed that this composition promotes a well-distributed, interconnected network, which enhances load transfer and energy dissipation while effectively suppressing delamination damage. In contrast, other compositions exhibited limited performance improvements due to phase separation or agglomeration. The phase structures of interlayers with different PPESK/PEEK ratios are presented in Figure 50.

**Figure 51:**
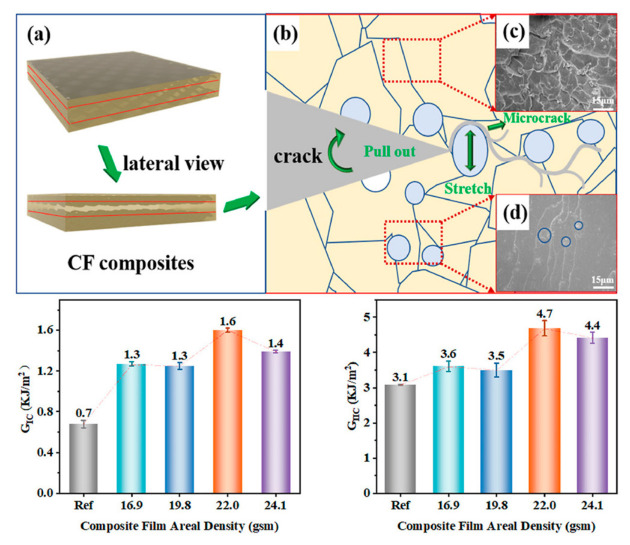
(**a**) EP layer between CF/EP composite layers, (**b**) schematic diagram of synergistic toughening mechanism of PEK-C and PES and SEM images of fracture surface of (**c**) EP resin blending with 10 phr PEK-C and (**d**) EP resin blending with 2 phr PES [185].

Similar phase structure concepts have also been demonstrated in thermoset laminates and provide useful mechanistic support for interlayer toughening. Xue et al. [185] prepared bicomponent PEK-C/PES porous films by phase inversion and used them as interlayers in CF/EP composites. The films remained stable at the epoxy infusion temperature of 100 °C, helping maintain resin permeability during infusion. During heating and curing at 180 °C, the films gradually dissolved into the epoxy matrix and formed a hybrid interlaminar morphology composed of flake-like PEK-C phases and sea-island-like PES phases. At the optimal areal density of 22.0 gsm, the Mode-I and Mode-II fracture toughness increased by 135.3% and 51.6%, respectively, and the compression-after-impact strength increased by 62.4%. A schematic illustration of the synergistic toughening mechanism and corresponding SEM images of the phase structure are shown in Figure 51.

**Figure 52:**
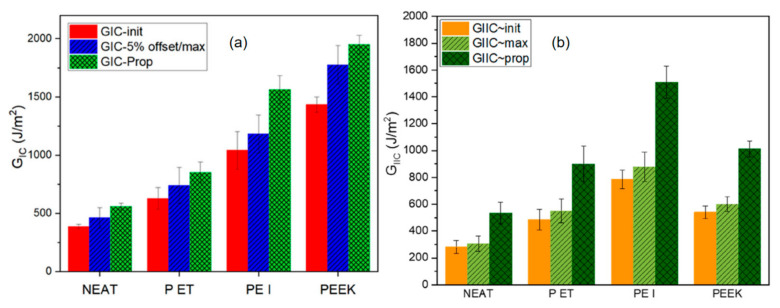
Mode-I fracture toughness results of neat, PET, PEI, and PEEK laminates. (**a**) Mode-I fracture toughness values. (**b**) Mode-II fracture toughness values [186].

Thermoplastic film interlayers further clarify the role of interlayer penetration, adhesion, and failure mode in fracture resistance. Too et al. [186] introduced PET, PEI, and PEEK thermoplastic films as interlayers into carbon fiber/epoxy composites via a melt infusion technique to enhance interlaminar properties. The results showed that the PEEK interlayer exhibited the most pronounced effect, with the initiation, propagation, and maximum values of Mode-I interlaminar fracture toughness (ILFT) increasing by 271%, 248%, and 284%, reaching 1435 J/m^2^, 1950 J/m^2^, and 1776 J/m^2^, respectively. The Mode-II ILFT also increased by 96% to 600 J/m^2^. A comparison of Mode-I and Mode-II fracture toughness for PET-, PEI-, and PEEK-modified laminates is presented in Figure 52. Fractographic analysis revealed that melt infusion enabled thermoplastic resin to penetrate between fiber bundles, forming a thermoplastic/epoxy/carbon fiber interlocked three-phase toughening region. This structure avoids the formation of weak interfaces typically associated with conventional interlayers and enhances energy dissipation through mechanisms such as fiber bridging, plastic deformation, and interfacial interlocking, thereby effectively extending crack propagation paths. Quan et al. [187] further demonstrated that interlayer/matrix adhesion is critical for activating these mechanisms. After 15 min of UV treatment, the water contact angle of ultra-thin PEEK films decreased from 84.1° to 30.45°, and the Mode-I and Mode-II fracture toughness of CF/epoxy laminates increased by up to 227% and 441%, reaching approximately 960 J/m^2^ and 6.15 kJ/m^2^, respectively. These studies indicate that interlayer toughening depends not only on the toughness of the inserted material, but also on its adhesion, penetration state, and crack-path regulation.

Functional interlayer design improves CFRTP performance mainly through local resin supplementation and interlaminar toughening. Powder or resin-rich interlayers can compensate for insufficient local resin contact and reduce interlaminar voids, whereas phase structured thermoplastic interlayers can enhance fracture energy dissipation by regulating crack paths and deformation modes. The main advantage of this strategy is that it directly targets the interlaminar region, where voids, weak bonding, and delamination are most likely to develop. Its limitations are also closely related to the localized introduction of an additional phase. If the interlayer is too thin or poorly distributed, the improvement in resin supply or toughening may be limited. If it is too thick, it may generate resin-rich regions, reduce the effective fiber volume fraction, and weaken fiber-dominated in-plane properties. Incompatible melting, dissolution, or crystallization behavior may restrict chain interdiffusion and create discontinuous interfaces. For phase-structured interlayers, the reinforcing effect is highly sensitive to phase morphology; discontinuous networks, particle agglomeration, or unstable phase separation can reduce load transfer efficiency and create new stress concentration sites. In addition, many thermoplastic-interlayer toughening studies are still based on CF/epoxy systems and should be used as mechanistic references rather than direct evidence for CFRTP manufacturing. Future studies should focus on interlayer thickness control, phase structure stability, processing reproducibility, fiber volume fraction retention, and long-term durability under thermal, mechanical, and environmental service conditions.

### 4.4. Critical Comparison and Application Boundaries of Interface Engineering Strategies

The preceding sections show that interface engineering strategies for CFRTP act at different structural scales. Sizing agent modification primarily regulates the carbon fiber surface, interfacial structural design adjusts the initial fiber–matrix distribution, and functional interlayer design targets the interlaminar region. These strategies are not mutually exclusive, but their effectiveness is limited by different processing and structural constraints. The main limitations and reprocessing/recycling impacts of the three strategies are summarized in Table 5.

The comparison further suggests that the selection of an interface engineering strategy should be problem-oriented rather than universally applied. When poor fiber–matrix adhesion is the dominant limitation, matrix-compatible sizing agents are the most direct and scalable option. When the main difficulty arises from the high melt viscosity of thermoplastic matrices and insufficient intra-bundle impregnation, structural redistribution of fibers and resin is more effective because it reduces the characteristic impregnation distance. When delamination or weak interlaminar bonding becomes the critical failure mode, functional interlayers can provide localized resin supplementation and additional fracture energy dissipation. In practical applications, these strategies may be combined, but excessive modification may increase process complexity, disturb fiber architecture, create resin-rich regions, or reduce the stability of recycled and reprocessed materials. Therefore, future interface engineering for CFRTP should shift from single-factor enhancement toward coordinated design, in which surface chemistry, resin distribution, and interlaminar toughening are optimized together while maintaining processability, mechanical reliability, and recycling compatibility.

## 5. Conclusions and Prospects

In summary, carbon fiber-reinforced thermoplastic composites have exhibited substantial potential as advanced lightweight structural materials owing to their high specific mechanical properties, impact resistance, weldability, reprocessibility, and potential recyclability. Compared with thermoset composites, the melt-processable nature of thermoplastic matrices provides important advantages for rapid forming, repair, and sustainable manufacturing. Nevertheless, the elevated melt viscosity of thermoplastic resins remains a central challenge, restricting resin flow, fiber impregnation, interfacial adhesion, and overall process robustness across nearly all forming methodologies. These difficulties are further intensified by reinforcement architecture and the chemical inertness of carbon fiber surfaces. Therefore, CFRTP manufacturing should be understood not as a single forming operation, but as a coupled materials–process–interface problem.

From a processing standpoint, each forming technique presents distinct advantages and application boundaries. Stamping and injection molding facilitate high-volume production of components, but they are still affected by defects such as wrinkling, fiber breakage, shrinkage, and warpage. Pultrusion and filament winding are well-suited for constant cross-section profiles and axisymmetric structures, respectively, yet they face difficulties in achieving complete fiber impregnation, stable consolidation, and precise tension control under high-speed conditions. Automated fiber placement offers high precision and design flexibility for large laminated structures, but its narrow thermal–mechanical process window requires strict control of heating, compaction, crystallization, and cooling. Additive manufacturing provides exceptional design freedom, but insufficient fiber impregnation, weak interlayer bonding, and limited forming efficiency still prevent its mechanical performance from reaching that of more established processes.

Interface engineering provides a complementary route to address the intrinsic limitations of high-viscosity thermoplastic systems. Chemical sizing can improve initial wetting by increasing carbon fiber surface energy and constructing a matrix-compatible interphase. Interfacial structural design, including fiber spreading, commingled yarns, and powder impregnation, can shorten effective resin penetration paths and improve resin distribution before consolidation. Functional interlayers can locally enhance interlaminar bonding, fill void-prone regions, and regulate crack propagation. These strategies are not mutually exclusive; instead, they should be integrated with forming processes to improve wetting, impregnation, load transfer, and damage resistance. However, their effectiveness remains dependent on coating uniformity, interlayer morphology, thermal stability, process compatibility, fiber volume fraction retention, and recycling or reprocessing behavior.

**Future research endeavors should prioritize the following avenues:**

Future research should place greater emphasis on the challenges that most directly restrict the industrial implementation of CFRTP technologies. The first priority is to improve manufacturing robustness. Although several forming processes have shown clear technical potential, their broader application still depends on the stable production of low-defect structures under realistic processing rates and component sizes. Future studies should therefore focus on widening the processing window, improving impregnation and consolidation consistency, and reducing quality fluctuations caused by thermal gradients, fiber movement, and non-uniform cooling.

A second priority is the development of material systems and intermediate forms designed for processability. For high-performance CFRTP, excellent thermal and mechanical properties alone are insufficient if the matrix remains difficult to melt, flow, impregnate, or consolidate within an industrial cycle. Low-melting thermoplastic matrices, commingled yarns, powder-impregnated preforms, and stable prepreg tapes are therefore important not only as material innovations, but also as routes to reduce processing severity and improve manufacturing efficiency. The key requirement is to enhance processability while retaining structural performance, durability, weldability, and reprocessing capability.

Interface engineering should also evolve from performance enhancement at the specimen scale toward process-compatible and scalable implementation. Sizing agents, fiber–matrix structural design, and functional interlayers can improve wetting, load transfer, and resistance to delamination, but their practical value depends on whether they remain stable during high-temperature forming and repeated consolidation. Future interface strategies should therefore be designed together with the selected forming process, so that improved interfacial bonding does not introduce new problems such as resin-rich regions, fiber architecture disturbance, or reduced recyclability.

Fourth, predictive modeling, in situ monitoring, standardized evaluation, and structural-level validation should be strengthened as enabling tools for industrial qualification. Process models and data-driven approaches can reduce trial-and-error optimization only when they are connected with realistic structural features such as voids, crystallinity, fiber orientation, residual stress, and interfacial bonding. Meanwhile, standardized testing methods are required to compare void content, impregnation quality, interlayer bonding, dimensional stability, durability, and recyclability across different material systems and forming routes. More importantly, validation should move beyond small coupons and simplified geometries toward large, thick, multidirectional, and service-relevant CFRTP structures. Such efforts are essential for establishing reliable design allowables, improving process certification, and promoting the transition of CFRTP manufacturing from promising laboratory demonstrations to robust industrial production.

Overall, the industrial development of CFRTP will depend on the coordinated progress of reproducible processing, processable materials, scalable interface engineering, and validated structure-level performance.

## Figures and Tables

**Table 1:** Comparison of representative reviews.

Reference	Main Topics Covered	Difference from the Present Review	Ref.
Chen et al.	Provided a focused review of thermo-stamping processes and simulation progress for continuous fiber-reinforced thermoplastic composites.	This review is not limited to a single process; rather, it compares multiple CFRTP forming routes and discusses their process-specific limitations and application boundaries.	[20]
Minchenkov et al.	Reviewed thermoplastic pultrusion, including process principles, materials, equipment, applications, and development challenges for continuous profiles.		[21]
Donough et al.	Reviewed process modeling of in situ consolidated thermoplastic composites by automated fiber placement, with emphasis on thermal, mechanical, and physical modeling approaches.		[22]
Periasamy et al.	Reviewed interfacial engineering methods for thermoplastic composites and analyzed strategies for enhancing fiber/matrix interfacial performance.	The present review discusses interface engineering not only as an interfacial strengthening method, but also as a practical strategy for improving wetting, impregnation, and interfacial bonding in high-viscosity CFRTP systems.	[23]
Wang et al.	Provided a broad and contemporary review of carbon fiber-reinforced thermoplastics, covering materials, manufacturing, post-processing, welding, applications, and future perspectives.	The present review complements this broad overview by focusing more specifically on processability challenges, comparative assessment of representative forming processes, and interface engineering for improving impregnation and bonding.	[24]

**Table 2:** Characteristic properties of CFRTP.

System	Crystallization Behavior	Glass Transition Temperature (Tg)/Melting Temperature (Tm)	Thermal/Chemical Resistance	Cost	Ref.
CF/PEEK	Semi-crystalline; carbon fiber accelerates crystallization; cooling rate strongly affects crystallinity	Tg ≈ 143 °C; Tm ≈ 344.8 °C	Excellent thermal and chemical stability; high long-term service temperature	Very high	[45]
CF/PPS	Semi-crystalline; carbon fiber and nucleating agents influence crystallization behavior	Tg ≈ 90 °C; Tm ≈ 280 °C	Good thermal and chemical resistance; relatively brittle	Medium	[46]
CF/PA6	Semi-crystalline; carbon fiber induces crystallization; fast crystallization rate	Tg ≈ 50 °C; Tm ≈ 224 °C	Moderate chemical resistance; pronounced moisture sensitivity	Low	[47]
CF/PEI	Amorphous; no crystallization; interfacial compatibility related to chain structure	Tg ≈ 215 °C; no Tm	Excellent thermal and chemical resistance; stable at elevated temperatures	High	[48]
CF/PES	Amorphous; no crystallization; enhanced interfacial compatibility	Tg ≈ 211 °C; no Tm	Excellent thermal and chemical resistance; suitable for high-temperature structural components	High	[49]

Note: The listed Tg and Tm values mainly reflect the thermal characteristics of the thermoplastic matrix phase. Carbon fibers generally have a limited influence on the nominal Tg and Tm values, but they may significantly affect crystallization kinetics, crystallinity, and crystalline morphology in semi-crystalline matrices [47,48].

**Table 3:** Comparative assessment of representative CFRTP forming processes.

Process	Applicable Form and Technology Maturity	Process Advantages and Limitations
Stamping	Mature; mainly uses prepreg sheets (e.g., fabric or unidirectional tape laminates), typically for medium-sized shell or semi-structural parts, such as battery housings and hat-section structures.	Enables short cycle times and high consolidation quality; however, it has limited adaptability to deep-draw structures and regions of high curvature, and is prone to wrinkling and springback.
Injection	Mature; mainly uses short/long fiber-reinforced thermoplastic pellets, typically for small-to-medium complex components requiring high efficiency and high dimensional accuracy.	Can form highly complex shapes and is suitable for mass production; however, fiber shortening and non-uniform orientation weaken mechanical properties, and the parts are prone to shrinkage and warpage.
Pultrusion	Medium maturity; mainly uses continuous fiber-reinforced thermoplastic prepreg tape/yarn (e.g., commingled yarns), typically for constant cross-section long profiles such as rods, beams, and stiffeners.	Enables continuous production with high material utilization; however, insufficient impregnation can occur at high pulling speeds, and transverse strength is very low, making the profile prone to splitting.
Filament Winding	Medium maturity; mainly uses continuous fiber unidirectional prepreg tape/yarn, typically for axisymmetric structures such as pressure vessels, pipes, and drive shafts.	The fiber path is highly controllable and enables jointless bodies of revolution; however, in dome or variable-curvature regions, the coordinated control of tension, heating, and consolidation is very difficult.
AFP	Low maturity; mainly uses continuous fiber unidirectional prepreg tape, typically for large laminated structures such as aircraft skins and stiffened panels.	Offers a high degree of automation and design flexibility; however, achieving low-porosity in situ consolidation quality remains difficult, and the equipment is extremely expensive.
AM	Low maturity; mainly uses continuous fiber-reinforced thermoplastic filament, primarily for prototypes, customized components, and locally reinforced structures.	Provides unparalleled design freedom and requires no molds; however, the finished parts suffer from weak interlayer bonding, and the printing speed is slow.

**Table 4:** General process–structure–property relationships in CFRTP forming.

Forming Parameter	Effect on Microstructure and Performance	Refs.
Forming temperature	Within an appropriate processing window, increasing temperature reduces matrix viscosity, promotes resin flow and fiber impregnation, and improves interlayer contact and interfacial bonding, thereby enhancing interlaminar properties and laminate integrity. Excessive temperature may cause matrix degradation, resin loss, or thermal damage, leading to reduced mechanical performance.	[146]
Heating rate/thermal history	Proper thermal history promotes uniform melting, stable resin flow, and consistent consolidation. Rapid or non-uniform heating may generate temperature gradients, incomplete melting, local overheating, uneven crystallization, and non-uniform interlayer bonding, resulting in unstable mechanical properties.	[117]
Dwell/consolidation time	Adequate dwell time allows resin infiltration, void escape, molecular interdiffusion, and interlayer fusion, thereby improving impregnation quality and interlaminar properties. Excessive dwell time may prolong the cycle and increase the risk of thermal degradation or residual stress accumulation.	[147]
Cooling rate	Cooling rate governs crystallinity, crystalline morphology, shrinkage behavior, and residual stress development. Controlled cooling helps balance stiffness, toughness, interlaminar properties, and dimensional stability, whereas excessively rapid or non-uniform cooling may induce crystallinity gradients, warpage, springback, or unstable mechanical performance.	[79,148]
Pressure/compaction force	Appropriate pressure promotes fiber bed compaction, intimate contact, void removal, and thickness uniformity, thereby improving consolidation quality, ILSS, compression performance, and dimensional consistency. Excessive pressure may cause fiber distortion, resin squeeze-out, or local fiber-rich regions, weakening structural uniformity.	[149]

**Table 5:** Comparative assessment of interface engineering strategies for improving wetting and impregnation in CFRTP.

Strategy	Main Limitation	Reprocessing/Recycling Impact
Sizing agent modification	Highly sensitive to sizing chemistry, coating uniformity, thickness, thermal stability, and long-term interfacial durability.	Good overall, but depends on the thermal stability and matrix compatibility of the sizing layer during repeated melting, welding, or reconsolidation.
Interfacial structural design	Requires uniform fiber–matrix distribution and stable fiber architecture; uneven commingling, powder distribution, spreading, or compaction may generate local dry spots or resin-rich regions.	Excellent when the same thermoplastic matrix is used, because this strategy mainly changes the initial spatial distribution of fibers and resin without introducing chemically incompatible phases.
Functional interlayer design	Excessive or non-uniform interlayers may create resin-rich regions, reduce effective fiber volume fraction, and weaken fiber-dominated in-plane properties; phase morphology must also be controlled.	Good when matrix-compatible thermoplastic interlayers are used, but repeated processing may alter interlayer thickness, phase morphology, or local resin distribution.

## Data Availability

No new data were created or analyzed in this study. Data sharing is not applicable to this article.
